# Correlating membrane‐protein dynamics with function: Integrating bioinformatics, molecular dynamics, and single‐molecule FRET

**DOI:** 10.1002/pro.70352

**Published:** 2025-10-23

**Authors:** Hugh R. Higinbotham, Christine A. Arbour, Barbara Imperiali

**Affiliations:** ^1^ Department of Physics Massachusetts Institute of Technology Cambridge Massachusetts USA; ^2^ Department of Biology Massachusetts Institute of Technology Cambridge Massachusetts USA; ^3^ Department of Chemistry Massachusetts Institute of Technology Cambridge Massachusetts USA; ^4^ Present address: Synthetic Molecule Design and Development (SMDD) Eli Lilly and Company Indianapolis Indiana USA

**Keywords:** glycoconjugate biosynthesis, membrane protein, molecular dynamics, non‐canonical amino acid mutagenesis, single‐molecule FRET, substrate specificity

## Abstract

We present a strategy that deploys structural bioinformatics, molecular simulation, and single‐molecule Förster Resonance Energy Transfer (FRET) microscopy for observing the ligand‐dependent conformational dynamics of integral membrane proteins in situ. We focus on representative members of the small monotopic phosphoglycosyl transferase (SmPGT) superfamily, which catalyze the transfer of a phosphosugar from a soluble nucleotide‐sugar donor to a membrane‐embedded polyprenol phosphate acceptor in the initiating step of glycoconjugate biosynthesis in prokaryotes. Substrate‐specific structural features were identified across the superfamily and correlated with ligand‐dependent conformational dynamics in all‐atom simulations. To experimentally validate the role of this motion in ligand binding, we developed a platform to monitor intramolecular protein dynamics in a native‐like lipid environment. The presented approach incorporates selective cysteine protein labeling and non‐canonical amino acid mutagenesis with bicyclononyne‐tetrazine click chemistry to assemble dual‐labeled variants of PglC, the initiating enzyme of the N‐linked protein glycosylation pathway from the *Campylobacter* genus. The modified proteins are solubilized in styrene‐maleic acid liponanoparticles (SMALPs), which provide a model membrane environment. The conformational changes of PglC upon inhibitor binding correlate with inhibitor potency. The single‐molecule FRET‐SMALP strategy can be adapted to investigate protein dynamics across the superfamily of SmPGTs with different substrate selectivity, where structure prediction and molecular dynamics support significant conformational changes upon ligand binding.

## 1 INTRODUCTION

Integral membrane proteins (IMPs) comprise approximately 25% of proteomes across kingdoms of life (Krogh et al., 2001). The specific composition and physicochemical properties of membranes vary widely, and the diverse interplay of this environment with IMP structure and function is a rich target for investigation (Brown et al., 2025; Levental & Lyman, 2023; Teo et al., 2019). Major advances in x‐ray crystallography and Cryo‐electron microscopy have led to the determination of many membrane protein structures (Nastou et al., 2020), and new membrane mimetic solubilization techniques are being introduced with increasing frequency (Autzen et al., 2019; Ayub et al., 2024; Biou, 2023; Choy et al., 2021; Kermani, 2021). Through the application of conformational restraints or advanced data processing, these techniques can also capture conformational variation in proteins in different “snapshots” and provide crucial information about their structural dynamics (Meszaros & Westenhoff, 2024; Wu & Rapoport, 2021; Zhong et al., 2021). However, despite these advances, the perturbations to IMPs necessary for sample preparation pose a challenge to connecting high‐resolution structural information to the native dynamics associated with functional enzyme activity. Methods that simulate and experimentally characterize protein conformational dynamics in biologically relevant membrane environments are therefore crucial to linking information from high‐resolution structural studies to the native physiological context of IMPs.

**FIGURE 1:**
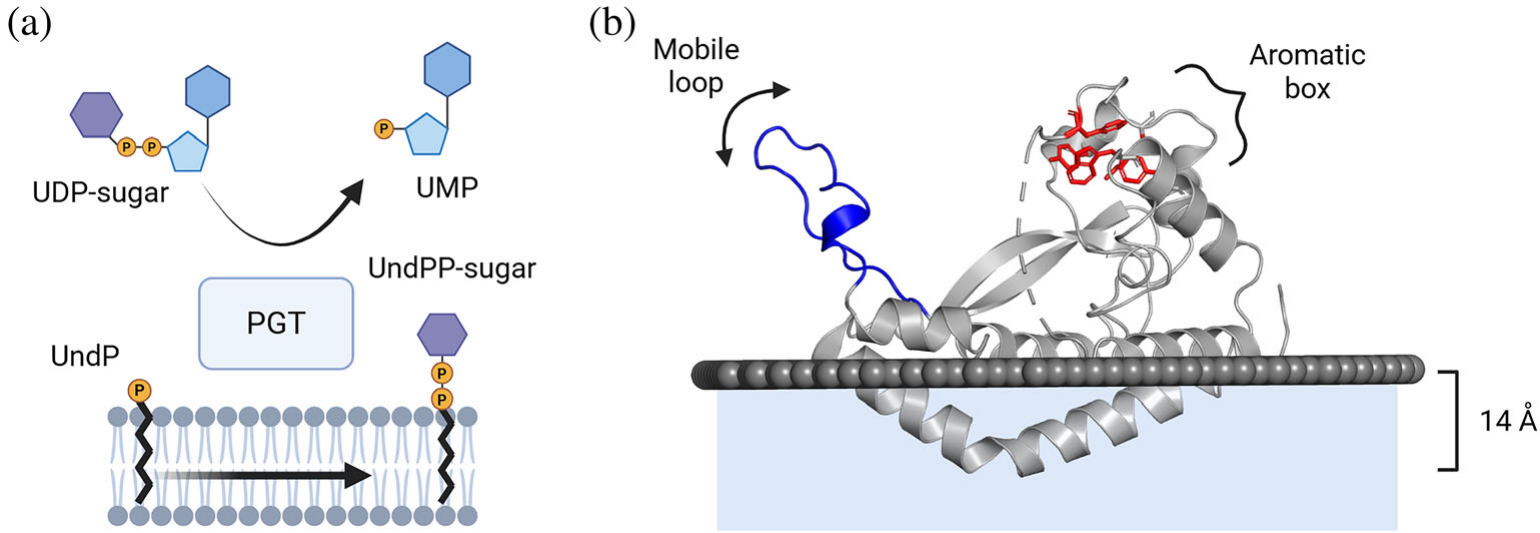
Reaction and dynamics of select small monotopic phosphoglycosyl transferase. (a) Schematic of the phosphoglycosyl transferase reaction. (b) Crystal structure of detergent‐solubilized *Campylobacter concisus* PglC with membrane interface modeled using the Orientations of proteins in membranes (server) (Lomize et al., 2011). Blue ribbon—residues 61–81—represents the mobile loop identified in Protein Data Bank (PDB ID): 8E37. Red residue side chains form the aromatic box motif identified in the refined structure PDB ID: 8G1N (Anderson et al., 2023), residues 184–193 are disordered and represented as a dashed gray loop.

IMPs in membrane‐associated glycan assembly pathways are of particular interest due to their essentiality in bacterial survival, host–microbe interactions, and pathogenicity (Costa & Iraola, 2019; Szymanski, 2022). These pathways function at the cytoplasmic face of cell membranes and represent key targets for developing new classes of antibiotics that inhibit the biosynthesis of selected virulence‐associated bacterial glycoconjugates. Critical to many glycoconjugate assembly pathways are phosphoglycosyl transferase (PGT) enzymes, which initiate glycan biosynthesis with a chemically distinct priming step. PGTs are categorized into two superfamilies distinguished by the membrane topology (monotopic or polytopic) of the minimal catalytic domain (O'Toole et al., 2021). The monotopic PGTs (monoPGTs) are exclusively prokaryotic and coordinate a divalent magnesium cofactor to catalyze phosphosugar transfer from a soluble nucleotide‐sugar donor to a polyprenol phosphate acceptor that anchors the forming glycoconjugate to the membrane (Figure 1a) (Das et al., 2017). Proteome‐wide bioinformatics and functional analyses have recently enabled the classification of small monoPGTs, representing the minimal catalytic subunit, into clusters that share a common UDP‐sugar substrate (Durand et al., 2024).

The small bacterial monoPGTs (SmPGTs) from the *Campylobacter* genus, known as PglCs, act on UDP‐di‐*N*‐acetyl‐bacillosamine and initiate a general N‐linked protein glycosylation pathway (Cain et al., 2020; Nothaft et al., 2012) (Table S1 and Figure S1). PglC from *Campylobacter concisus* (*Cc* PglC) is the only SmPGT with an experimentally determined structure (Figure 1b), and it remains one of only two experimental structures of any monoPGTs (Dodge et al., 2024; Ray et al., 2018). Variations between the PglC monomers in an x‐ray structure that captures eight polypeptides in the octameric asymmetric unit and analysis of the position of a mobile loop (residues 61–81) correlate with molecular dynamics (MD) simulation in all‐atom bilayer systems. However, the connection between these structural dynamics and monoPGT function remains unclear (Entova et al., 2018; Majumder et al., 2023). The *Cc* PglC structure was recently updated to resolve additional C‐terminal residues and identifies a conserved aromatic box motif that anchors the C‐terminus in place (Figure 1b) (Anderson et al., 2023). With both a validated full‐length structure of *Cc* PglC and vastly expanded sequence information across the superfamily of SmPGTs, we can now investigate structural dynamics across different UDP‐sugar substrate‐specific families (Durand et al., 2024). Further investigation of the PGT conformational dynamics associated with substrate and ligand binding, conducted at the membrane interface, requires a targeted approach that integrates computational and experimental approaches.

Herein we report a combined computational and experimental strategy for investigating membrane protein dynamics in the presence of small molecule ligands to trigger and observe conformational changes. In this approach, we employ SmPGTs from the *Campylobacter* genus, including the *Cc* PglC and the *Campylobacter jejuni* (*Cj*) PglC (Table S1 and Figure S1). The *Cj* PglC is derived from a clinically relevant strain that has been the focus of inhibitor development due to its association with gastroenteritis (Acheson & Allos, 2001). In the presence of nucleoside analog inhibitors, we observe correlated closure of a mobile loop with competitive inhibition of the UDP‐sugar substrate, which is a proxy for binding affinity. By combining information from structural bioinformatics, MD simulations, and single‐molecule Förster Resonance Energy Transfer (smFRET) microscopy, we can assemble a clearer picture of mono PGT‐substrate interactions, which will corroborate and aid the design of future structural studies and small molecule inhibitor discovery campaigns.

## 2 RESULTS

### 2.1 Computational evidence of ligand‐dependent loop mobility

**FIGURE 2:**
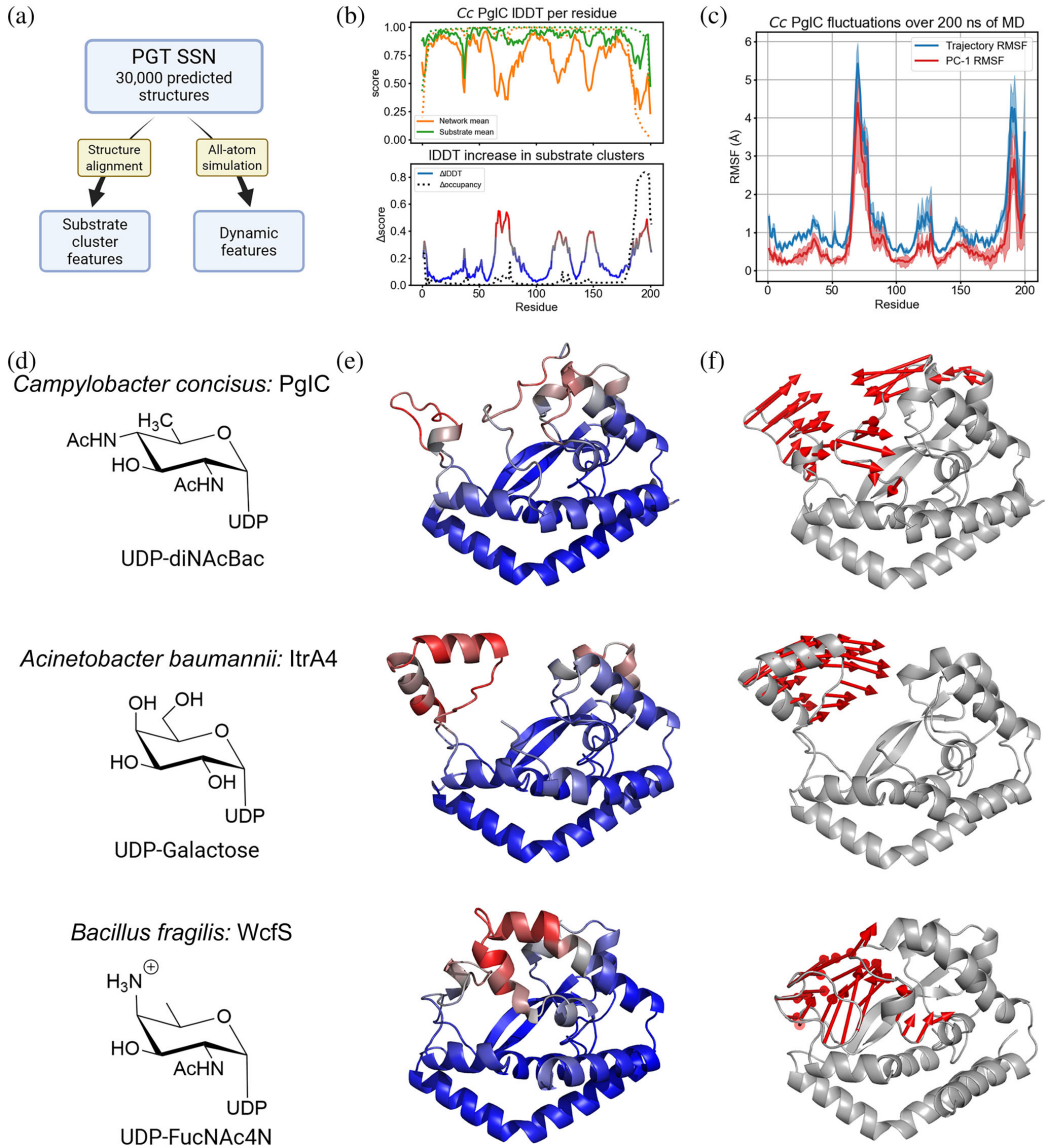
Substrate‐specific structural motifs and associated dynamics. (a) Analysis schematic of predicted structures from the phosphoglycosyl transferase (PGT) sequence similarity network. (b) *Top*: Average lDDT in pairwise structure alignments between *Cc* PglC and other PGTs in the sequence similarity network (SSN) (Durand et al., 2024). Orange shows the network average and green shows only the diNAcBac cluster. Dotted lines show PGT occupancy at a given residue. *Bottom*: lDDT increase within substrate clusters by residue. Dotted line shows increase in occupancy. (c) Root‐mean‐square fluctuation (RMSF) by residue of *Cc* PglC over 200 ns of molecular dynamics (MD) simulation. Blue indicates total fluctuation while red shows the first principal component, and shaded regions indicate the variation between three replicate runs. (d) PGT identity and cognate sugar substrate. (e) AlphaFold2 structure predictions colored by average lDDT increase in substrate‐specific clusters per residue. (f) Visualization of the first principal component of each PGT backbone from one of the three simulations.

We first sought to identify key dynamic features across the SmPGT superfamily. SmPGTs catalyze the transfer of a phospho‐sugar to undecaprenol phosphate (UndP) in the presence of a divalent magnesium cofactor (Figure 1a,b). SmPGTs are highly selective for their cognate nucleotide diphosphate sugar substrate, which has been demonstrated in a recently published SmPGT sequence similarity network (SSN), which comprised approximately 30,000 unique protein sequences and was complemented by biochemical activity analysis (Durand et al., 2024). To investigate the SmPGTs, we compared patterns of structural variations across the SSN to the simulated dynamics of representative proteins from different substrate‐specific clusters (Figure 2a). The average local distance difference test (lDDT) score per residue was computed from pairwise 3D structural alignments using TM‐score (template modeling score) alignment (TM‐align), both between members of substrate‐specific clusters and between cluster representatives and the entire SmPGT network (Figure 2b). To probe SmPGT protein dynamics, we conducted all‐atom MD simulations of predicted structures of cluster representatives in protein‐bilayer systems. We identified dynamic features by comparing the root‐mean‐squared fluctuations (RMSF) and the dominant motion in principal component analysis (PCA) binned by amino acid residue (Figure 2c).

These dynamic features were then compared to substrate‐specific structural features of models from AlphaFold2 predictions from the SmPGT SSN. We conducted this analysis for three representative SmPGTs that act on different UDP‐sugar substrates, namely: *Cc* PglC (UDP‐diNAcBac), *Acinetobacter baumannii (Ab)* ItrA4 (UDP‐Gal), and *Bacillus fragilis (Bf)* WcfS (UDP‐FucNAc4N) (Figure 2d). Analogous regions of the three PGTs align closely within each substrate cluster relative to PGTs across the network (Figures 2e and S2a,c). Each PGT also features a dominant first principal component in which an analogous mobile element (ranging from 20 to 30 residues in length) shows the highest degree of concerted motion (Figures 2f and S2b,d). The most dynamic features in simulation closely correlate with those that are structurally conserved, specifically within a given substrate‐specific cluster (Figure S2e,f). This observation suggests that dynamic structural motifs of small PGTs across the superfamily evolved to better recognize distinct UDP‐sugars and catalyze phosphosugar transfer.

**FIGURE 3:**
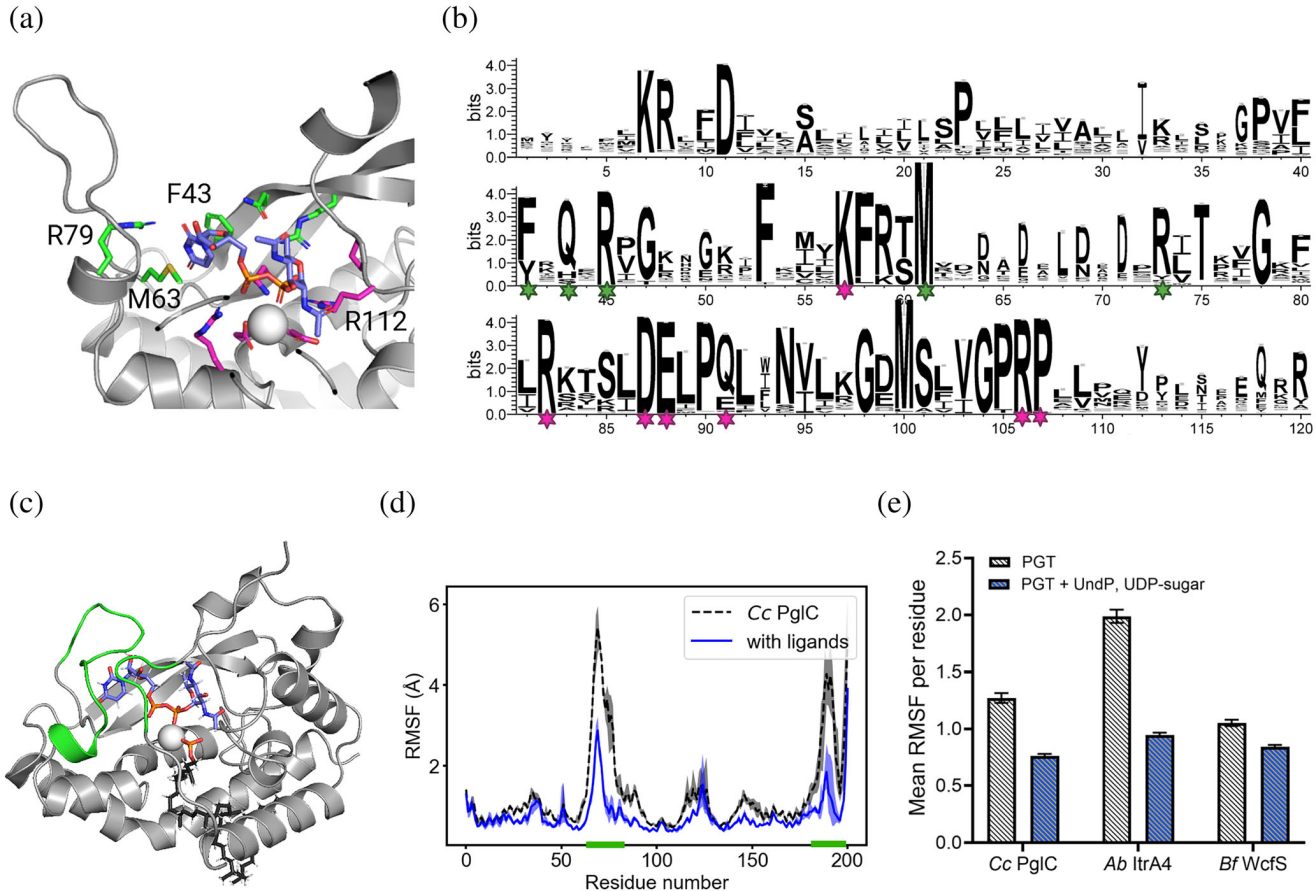
Phosphoglycosyl transferase (PGT) stabilization with ligands. (a) Active site closeup of Chai‐1 predicted pose of *Cc* PglC with UDP‐diNAcBac. Highlighted residues are conserved across the small monotopic phosphoglycosyl transferase (SmPGT) superfamily, with magenta showing previously known residues of the catalytic core and green showing newly identified residues. (b) Sequence logo plot of the SmPGT network with conserved residues from (a) highlighted. Only residues with >50% occupancy are shown to condense the scale of the plot. (c) Structure of *Cc* PglC with UDP‐diNAcBac and undecaprenol phosphate (UndP) after 500 ns simulated in a bilayer. Green highlights show significant structural rearrangement from the initial Chai‐1 prediction. The acceptor substrate UndP is shown in black sticks and the Mg^2+^ cation is shown as a gray sphere. (d) Average root‐mean‐squared fluctuations (RMSF) per residue for *Cc* PglC simulated in a bilayer with or without ligands. The mean value per residue from triplicate simulations of 200 ns is shown with shading that indicates the minimum and maximum values. (e) Mean RMSF per residue for substrate‐specific motifs with and without ligands. Simulations were initialized with Chai‐1 predicted complexes for the three exemplar PGTs.

To investigate the details of the UDP‐sugar binding pocket, we next conducted MD simulations with the cognate sugar substrate, magnesium cofactor, and UndP present. We used Chai‐1 to generate an initial docked structure of *Cc* PglC with its ligands, which reliably places the Mg^2+^ and UndP consistent with the crystal structures and prior simulations (Chai Discovery Team et al., 2024; Majumder et al., 2023). Although the UDP‐sugar binding site is unknown, a number of key residues conserved across the SmPGT superfamily are understood to interact with the UDP‐sugar substrate (Das et al., 2017). These residues are also predicted to interact with the diphosphate moiety and coordinate the magnesium ion in Chai‐1 structures (Figure 3a,b, magenta residues). Intriguingly, although Arg112 was originally proposed to interact with the uracil moiety (Entova et al., 2018), the UDP‐sugar is now predicted in the opposite orientation with Arg79 instead performing a similar role in uracil binding. Of particular note is the interaction of a highly conserved methionine and an aromatic residue of previously unknown function with the uracil group (Met63 and Phe43 for *Cc* PglC) that stabilizes the bound nucleobase and anchors the position of the mobile loop. We see highly consistent ligand placement for other SmPGTs as well (Figure S3). For both *Cc* PglC and *Ab* ItrA4, Chai‐1 predicts a more closed protein conformation of the substrate‐specific mobile loop when a UDP‐sugar is present, although *Bf* WcfS remains relatively closed in all Chai‐1 predictions (Videos [Link], [Link]).

We next conducted all‐atom MD with and without UndP and UDP‐sugar substrates present in a phospholipid bilayer, initialized with the corresponding Chai‐1 predicted structure. In all cases, the presence of substrates in this predicted orientation significantly stabilized the most dynamic residues that we have shown to be substrate‐specific (Figures 3d,e and S3). Of note is that *Cc* PglC still showed significant variation in the position of its mobile loop in the initial 200 ns simulations with substrates. Extending this condition to a full microsecond of simulation time showed a significant conformational rearrangement of this loop to close the active site around UDP‐diNAcBac (Figure 3c, Video S7). Taken together, these results suggest that Chai‐1 is able to reasonably predict the closed conformation of *Ab* ItrA4 and *Bf* WcfS when bound to their respective UDP‐sugar substrates but could not fully capture the more dramatic structural rearrangement of *Cc* PglC. Nevertheless, the Chai‐1 predictions enabled us to improve hypothesized PGT binding poses and substantially reduce the search space of poses and interactions using demanding MD simulations. Critically, the analysis also identified substrate‐protein interactions that could not have been determined otherwise due to challenges with the determination of high‐resolution crystal structures of the unusual monotopic protein.

### 2.2 Experimental platform to observe ligand‐dependent conformational changes

To investigate the predicted SmPGT dynamics in a physiologically relevant membrane context and its dependence on the presence of bound ligands, we developed a platform for labeling and immobilizing a PGT in a native‐like liponanoparticle for smFRET. Here, we focused on the SmPGT from the N‐linked glycosylation pathway of *C. jejuni*, *Cj* PglC. Compared to *C. concisus*, *C. jejuni* is a more pathogenic strain of *Campylobacter* responsible for the majority of infections from the genus (Costa & Iraola, 2019). *Cc* and *Cj* PglC are both highly active in vitro and specific to UDP‐diNAcBac, have 71.6% sequence identity and extremely similar AF predicted structures (Figure S1) which are in turn <1.51 Å root mean square deviation (RMSD) from the experimental crystal structure of PglC from *C. concisus* (Anderson et al., 2023). Additionally, *Cj* PglC has previously been evaluated against a library of nucleoside analogs developed as potential inhibitors (Durand et al., 2024). Therefore, *Cj* PglC is a valuable test subject where orthogonal approaches to determine ligand binding can better inform the design and development of inhibitory small molecules.

**FIGURE 4:**
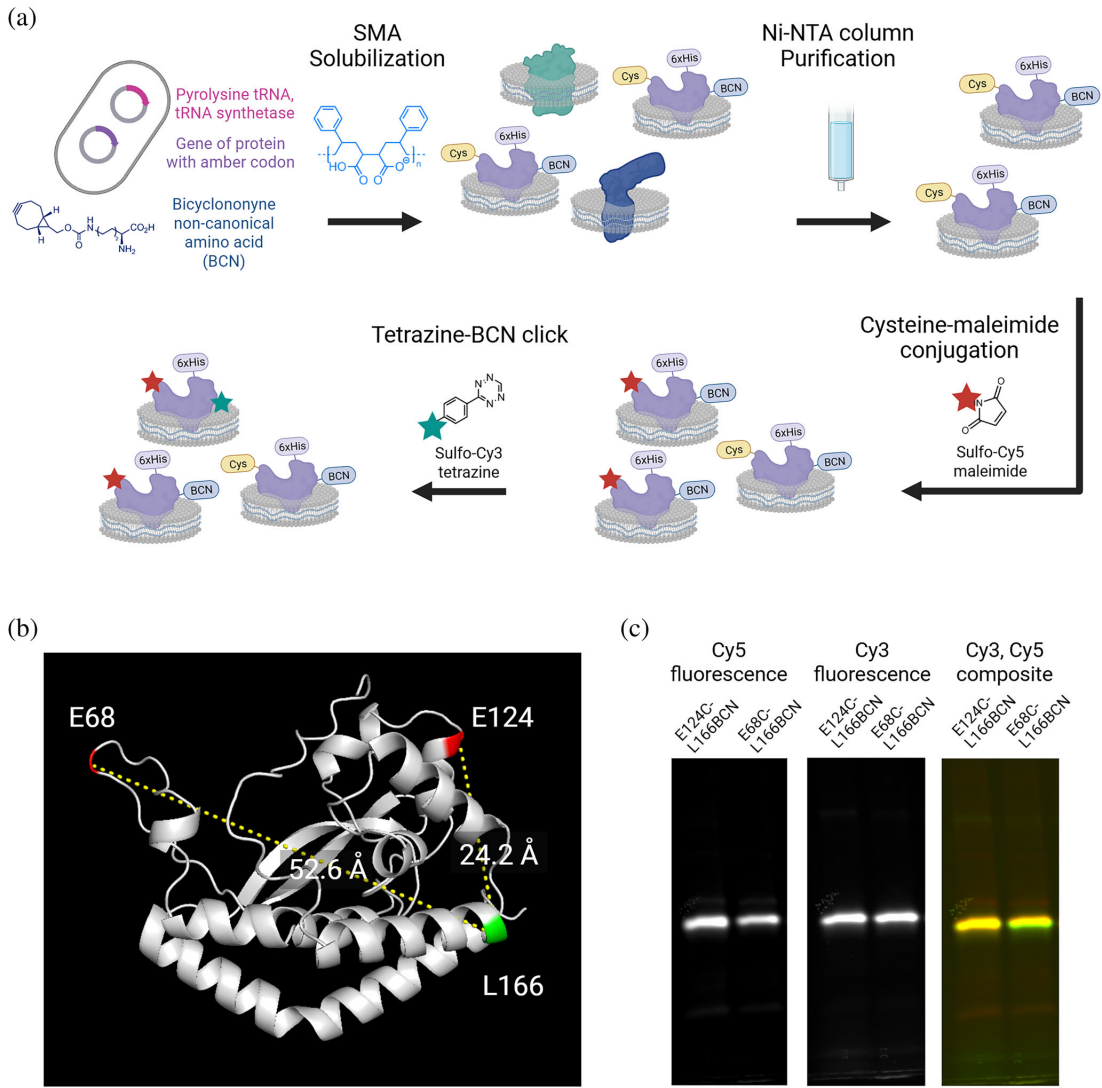
Solubilization and labeling of *Cj* PglC. (a) Schematic showing protein expression, solubilization, and sequential dual labeling in SMA liponanoparticles. (b) AF structure of *Cj* PglC showing residue sites for fluorophore conjugation in loop‐labeled variant (E68C‐L166BCN) and positive control (E124C‐L166BCN). (c) Fluorescence gel showing both dual variants purified in styrene maleic acid liponanoparticles and labeled with Cy3 and Cy5 fluorophores. Gel was imaged on a ChemiDoc MP system using 577–613 and 675–725 nm emission filters, respectively. BCN, bicyclononyne‐lysine.

We established a detergent‐free purification pipeline with orthogonal labeling of two specific residues with fluorescent dyes for single‐molecule FRET (Figure 4). Although similar detergent‐free solubilization and site‐specific labeling techniques have been utilized for FRET studies (Ponzar & Pozzi, 2023; Quast et al., 2019; Wang et al., 2014), to our knowledge this has not previously been conducted to study small membrane proteins in SMALP and thus required extensive development and validation. For the site‐specific introduction of Cy3 and Cy5 fluorophores for FRET, we deployed two efficient orthogonal conjugation strategies to minimize heterogeneity in the sample. As *Cj* PglC has no native cysteine residues, we applied site‐directed mutagenesis to incorporate cysteine at various sites for thiolate‐targeted alkylation with a sulfoCy5 maleimide derivative. For Cy3 incorporation, we applied non‐canonical amino acid mutagenesis using the pyrrolysine system (Wan et al., 2014), with an engineered transfer ribonucleic acid (tRNA) synthetase/tRNA^TAG^ pair for bicyclononyne‐lysine (BCN) incorporation at the amber stop codon (TAG) (Bartoschek et al., 2021; Mukai et al., 2015; Swiecicki et al., 2020). Both Cys and BCN incorporation sites were chosen at positions of low conservation across SmPGTs. The BCN sites were further screened for suppression in the absence of BCN (Figure S4a), from which we selected residue L166 for non‐canonical amino acid mutagenesis. We selected two dual variants: one with mutagenesis sites at E68 and L166 to measure the average position of the mobile loop, and one with modification at E124 and L166, which are significantly below the Förster radius of the Cy3‐Cy5 FRET pair to provide a positive control in a similarly labeled protein SMALP in our microscope system (Figure 4b). Both variants were overexpressed in a strain of *Escherichia coli* without specific assignment to the amber stop codon, solubilized from the cell envelope fraction into styrene‐maleic acid liponanoparticles (SMALPs), purified with a Ni‐NTA pulldown (Figure S4b), and sequentially labeled with sulfoCy5 maleimide and Cy3‐tetrazine (Figure 4c). This process ensured a native‐like membrane environment for in vitro measurements while eliminating intermediate detergent solubilization, which can be highly variable and deleterious to enzyme activity and stability (Arbour et al., 2023). Dual‐labeled SMALPs were subsequently run on a size exclusion column to remove excess dye and any remaining SMA or off‐target SMALPs (Figure S4c,d). Activity was confirmed for dual variants in SMALP using uridine 5'‐monophosphate UMP‐Glo™ (Figure S4e), and a radioactivity‐based activity assay, which established the compatibility of *Cj* PglC in the presence of the oxygen scavenging system used to reduce fluorophore photobleaching and triplet‐state quenching in the imaging buffer (Figure S4f) (Arbour et al., 2023; Das et al., 2016).

For Total Internal Reflection Fluorescence (TIRF) microscopy, we used a dual‐camera setup with a dichroic mirror splitting at 640 nm (Figure S5a). To calibrate the system for smFRET, we used Cy3 and Cy5 labeled DNA oligos tethered to PEG‐biotin‐coated glass coverslips for camera registration and *γ* correction (Figure S5b‐f). To tether the protein‐SMALP assemblies to the glass surface, we prepared PEG‐modified glass coverslips with a low density of polyethylene glycol nitrilotriacetic acid (PEG‐NTA) instead of PEG‐biotin (Figure S6a). We observed a high level of specificity for SMALPs whether a His_6_‐tag or a Strep‐II tag was used for protein solubilization (Figure S6b), and an excess of soluble protein with a His_6_‐tag was unable to compete for these surface binding sites (Figure S6c). This suggests that the interactions between styrene maleic acid (SMA) in these liponanoparticles and the nickel nitrilotriacetic acid (Ni‐NTA) on the surface are sufficient to reliably attach solubilized protein and show enough avidity in this geometry to supersede His_6_‐tag anchoring. These findings are consistent with the known sensitivity of SMALPs to divalent cations in solution, as well as a tendency toward a small level of nonspecific interaction with Ni‐NTA that requires additional low imidazole washes during purification (Pollock et al., 2018). We hypothesize this to be a result of the negative charge of the SMA polymer causing interaction with nickel cations. This illustrates Ni‐NTA can be used to anchor SMALPs to glass slide surfaces directly, greatly simplifying the experimental design of protein constructs for surface‐tethered imaging experiments or binding arrays.

**FIGURE 5:**
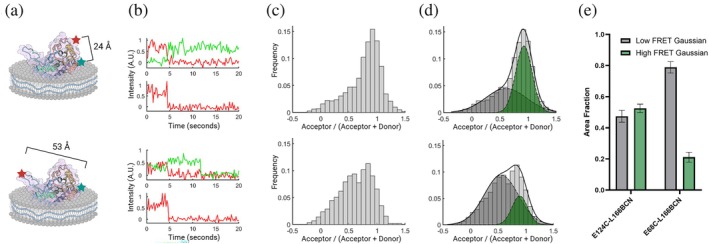
Analysis of Förster Resonance Energy Transfer (FRET) from dual‐labeled *Cj* PglC variants. (a) Labeling scheme of control variant vs. loop variant with associated α‐carbon to α‐carbon distances. (b) Sample intensity traces. Top panel is under 532 nm illumination, where green is Cy3 emission and red is Cy5 emission. Bottom panel is 640 nm excitation, taken in alternating frames. (c) FRET distributions over a larger dataset for each variant (*N* = 110 and 74, respectively). (d) The kernel density estimation of each distribution from (c) is fit with two Gaussians to show relative proportion of saturating and sub‐saturating FRET in the ensemble. (e) Area fraction of Gaussian fits by labeling variant. Error bars are set by the 95% confidence interval of fitting.

To validate the robustness of the purification and labeling platform for smFRET imaging, we first compared the average FRET ratio observed between the loop‐labeled variant E68C‐L166BCN with the high‐FRET control variant E124C‐L166BCN (Figure 5a). For each condition, videos were taken in a single batch under identical conditions. To confirm the presence of both fluorophores, we alternated excitation between donor and acceptor every frame and examined the population that showed donor emission recovery upon acceptor photobleaching while maintaining a near‐constant total emission intensity (*γ* correction factor near unity) (Figure 5b). In this way, we filtered single‐molecule traces to an ensemble of approximately 50–100 traces per condition. We examined the distributions of the FRET proximity ratio (Figure 5c). The control variant showed a dominant peak at saturating values of FRET where donor emission was undetectable relative to noise until acceptor bleaching. In contrast, the loop variant featured a smaller saturating peak as well as a broader distribution of intermediate FRET values. This bimodal distribution indicates the presence of an additional state where the mobile loop is in a closed configuration, closer to the main soluble domain of the protein. To assess the relative frequency of this state in the distribution, we fit the distribution to the sum of two Gaussians (Figure 5d). Note that the control distribution is not perfectly fit by a single Gaussian distribution due to a small proportion of lower FRET traces. Therefore, we quantify the relative proportion of saturating FRET as a comparative measurement describing how well we can distinguish FRET transfer at intermediate and close distances (Figure 5e).

**FIGURE 6:**
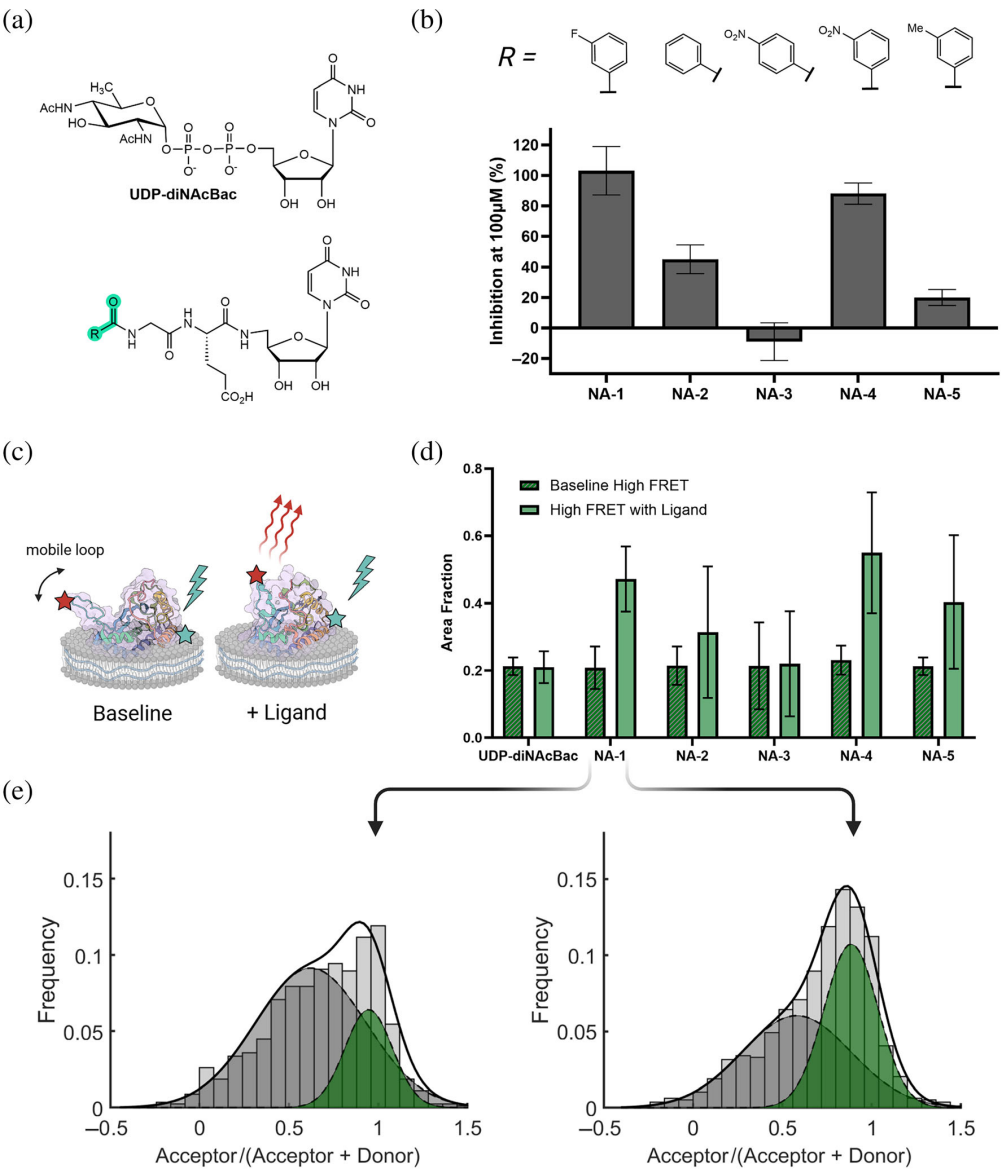
*Cj* PglC mobile loop closure in the presence of synthetic nucleoside analogs. (a) *Top*: Chemical structure of UDP‐diNAcBac. *Bottom*: Nucleoside analog (NA) scaffold structure. Variable R group highlighted in green. (b) *Top*: Variable R group for panel of five NA inhibitors. *Bottom*: Percent inhibition of *Cj* PglC. Activity was measured via UMP‐Glo with and without ligand to calculate percent inhibition. (c) Illustration of protein styrene‐maleic acid liponanoparticles in baseline and test conditions. (d) Fraction of high‐Förster Resonance Energy Transfer (FRET) observed in distribution of single‐molecule traces before and after addition of ligand. (e) *Left*: Control FRET ratio distribution and dual‐Gaussian fits of loop‐labeled variant before addition of NA‐1. *Right* FRET ratio distribution after addition of NA‐1, showing increased occurrence of high‐FRET state.

We proceeded to observe potential changes in the FRET distribution upon the addition of small molecule ligands. We used nucleoside mimetic compounds designed to be non‐hydrolyzable analogs of the UDP‐sugar substrate. This peptido‐nucleoside backbone was selected since this scaffold had produced the best inhibition for PglC to date through a competitive inhibition mechanism (Madec et al., 2018). To test the impact of substituted aryl groups on the sugar‐interacting region, different functionalized aryl groups were installed at the terminal portion of the compound, which resulted in a variety of inhibitory properties (Figure 6a,b) (Arbour & Imperiali, 2022; Durand et al., 2024). From this small series, the best inhibitors of *Cj* PglC are NA‐1 and NA‐4, with *K*_i_ values of 5.7 ± 1.3 and 9.7 ± 0.7 μM respectively, based on the UMP‐Glo assay and which compete for the UDP‐sugar binding site (Figure S7). To evaluate the effect of each compound on the closure of the mobile loop, we measured the distribution of FRET observed from a population of loop‐labeled *Cj* PglC SMALPs before and after the addition of a given ligand. This was done to check for consistency of the control distribution when imaging on different days.

In these analyses, we did not observe a significant difference in FRET when adding the cognate uridine 5'‐diphosphate (UDP)‐sugar substrate for *Cj* PglC; however, the nucleoside analogs show a significant correlation between percent inhibition and closure of the mobile loop (Figures 6c–e, S8). This suggests that the time scale at which UDP‐diNAcBac reacts to form the covalent intermediate is faster than the highest frame rate at which we can measure sufficient signal to distinguish different average loop positions (about 2.5 frames per second). As a class of competitive inhibitors, the percent inhibition of the non‐hydrolyzable analogs is a proxy for binding affinity to the active site. The correlation between high FRET and inhibition, therefore, indicates that closure of the mobile loop correlates with ligand binding, and that tighter binders will result in a higher frequency of the closed conformational state that can be observed in the distribution of smFRET traces.

## 3 DISCUSSION

### 3.1 Integrating computational and experimental techniques informed by the membrane environment

As more is discovered about the role of local membrane composition on the dynamics of membrane proteins, characterization techniques will need to account for local bilayer chemistry and geometry (Levental & Lyman, 2023). This is especially vital in the era of ubiquitous protein structure prediction and emerging advances in molecular docking and complex prediction based on large scale machine learning (ML), where precise details of the information used are obscured within a sea of bioinformatic and structural training data. To complement top‐down efforts to characterize the information used by ML structure prediction models (Zhang et al., 2024), cross‐validation that explicitly accounts for the physicochemical effects of membranes on protein structure and function is vital. The methodology developed in this work demonstrates the cross‐validation of ML‐based structural bioinformatics with MD, biochemical characterization, and smFRET microscopy to study protein dynamics in the context of phospholipid bilayers systems. For proteins like the SmPGTs that are exceptionally challenging to characterize structurally, it is critical to know what level of significance to assign different protein–ligand complex predictions.

### 3.2 PGT conformational dynamics are connected to UDP‐sugar binding

Our combined bioinformatic, computational, and experimental characterization of *Cj* PglC and close homologs independently correlates dynamics of the substrate‐specific SmPGT mobile loop to the binding of soluble ligands and indicates a closed conformational bound state.

The Chai‐1 predictions utilized in this study identified a uridine binding motif that required partial closure of the SmPGT mobile loop for repositioning amino acid side chains (namely Phe36, Met61, and Arg73 in Figure 2b). The ability to predict binding poses with conformational changes is an advantage for these emerging computational methods, but proteins like PglC illustrate that more dramatic structural dynamics may necessitate all‐atom MD and smFRET measurements to identify. The presence of correlated dynamics across the SmPGT superfamily indicates that structural flexibility may facilitate the evolution of new substrate specificities, as has been observed for broadly neutralizing antibodies (Ovchinnikov et al., 2018).

Screening with synthetic nucleoside analog inhibitors allows us to demonstrate that closure of the mobile loop plays a role in binding nucleoside analogs in the UDP‐sugar active site. The fact that the analogs are designed as substrate mimics that cannot be hydrolyzed allows corroboration on the role of the conformational changes on UDP‐sugar binding. In this study, we did not achieve sufficient sensitivity and temporal resolution to measure kinetics of the closed and opened states, but the clear correlation of high FRET with better binding competitive inhibitors corroborates the simulated dynamics of UDP‐sugar binding.

This work lays the foundation for future mutagenesis studies and single‐molecule kinetics to identify residues responsible for ligand specificity and further characterize the bound state, as well as further computational studies that generalize to the superfamily of SmPGTs. As more potent inhibitors are generated, they can be evaluated with this workflow to identify protein–ligand interactions.

### 3.3 Modular design of competitive inhibitors for specific protein–ligand interactions

The purification, labeling, and surface‐tethering platform allows us to use single‐molecule FRET microscopy to visualize conformational changes in small membrane proteins in situ. When combined with a panel of small molecule ligands, we can also identify ligand‐specific conformational changes. The SmPGTs and the library of UDP‐sugar analog inhibitors are an ideal test case to study the limits of complex prediction accuracy, as these proteins are small enough to be computationally tractable for a variety of computational approaches and substrate‐specific enough that individual changes in stereo‐ and/or regiochemistry can dramatically affect binding. Additionally, substrate‐mimetic nucleoside analog inhibitors allow direct comparison to UDP‐sugar binding, and the modular design streamlines force‐field optimization for further MD studies. Specific interactions may then be independently tested for binding and the induction of a conformational change by comparing nucleoside analog chemistry with our pipeline and targeted mutagenesis studies with data from the smFRET platform. Ligands can be screened for inhibition and induced fit by smFRET to streamline selection for structural studies to capture bound conformations.

## 4 MATERIALS AND METHODS

### 4.1 Protein structure prediction and network analysis

AlphaFold 2 structures for the SSN of SmPGTs were downloaded from the AlphaFold2 database if present (Jumper et al., 2021) or else predicted using LocalColabFold v1.5.5 (Mirdita et al., 2022) run with flags –amber, –templates, –num‐recycle 3, –use‐gpu‐relax on an NVIDIA GEFORCE RTX 2080 GPU. AlphaFold2 structure predictions of the PGT SSN were aligned and analyzed using analysis tools from the Foldseek repository. Example PGT structures were queried against the entire PGT network and aligned using Tmalign, and the per‐residue lDDT score was calculated. The average values were calculated in both the network and the substrate‐specific clusters to identify substrate‐specific structures.

Chai‐1 v0.4.0 predictions were run locally using an NVIDIA GEFORCE RTX 3070 GPU using the Colabfold MSA server and flags –num‐trunk‐recycles 3, –num‐diffn‐timesteps 200, –num‐diffn‐samples 5, –num‐trunk‐samples 1. Ligand‐bound complexes were predicted with Mg^2+^, UndP, and the cognate UDP‐sugar substrate using their simplified molecular input line entry system representations (Table S3). The top five ranked structures of *Cc* PglC with no ligands had RMSD <2.22 Å of either crystal structure and RMSD <0.83 Å of the AlphaFold 2 database predicted structure (Table S2). Given the consistently high similarity to the known *Cc* PglC structure and the lack of structures for other SmPGTs, we opted to predict five structures and select the top ranked model per condition to prepare systems for simulation.

### 4.2 All‐atom MD simulations

The procedure for all‐atom simulation of SmPGTs in membranes was adapted from recent work simulating the truncated structure of *Cc* PglC (Majumder et al., 2023). Briefly, protein and lipid bilayer systems were prepared using the CHARMM‐GUI webserver and simulated in the CHARMM36m force field (Huang et al., 2017; Wu et al., 2014) using GROMACS version 2024.3 (Abraham et al., 2015; Berendsen et al., 1995). Chai‐1 predicted structures were run through the PPM 2.0 server to initialize the location of the membrane interface (Lomize et al., 2011). The CgenFF webserver was used to generate topologies for UndP and UDP‐sugars (Vanommeslaeghe et al., 2010; Wu et al., 2014). Protein or protein–ligand complexes in membrane systems were built using the replacement method into a lipid bilayer composed of 200 total phospholipid molecules, specifically 67 mol% 1‐palmitoyl‐2‐oleoyl‐sn‐glycero‐3‐phosphoethanolamine (POPE), 23 mol% 1‐palmitoyl‐2‐oleoyl‐sn‐glycero‐3‐phosphoglycerol (POPG), and 10 mol% cardiolipin (CL) of defined acyl‐chain composition. The combined systems were solvated using the transferable intermolecular potential with three points water model with 0.15 M KCl. The membrane systems were then equilibrated in three replicate runs for at least 50 ns of simulation in the Isothermal‐isobaric (ensemble) with the Parrinello‐Rahman barostat and Nose‐Hoover thermostat via the CHARMM‐GUI protocol. Production simulations were run at a temperature of 303 K. Analyses were conducted either in GROMACS or with code written using the MDAnalysis Python package (Gowers et al., 2016; Michaud‐Agrawal et al., 2011).

### 4.3 Protein expression

Wild‐type and single‐cysteine variants of PglC were expressed with a C‐terminal His_6_‐tag or with a Strep‐Tag and in vivo SUMO tag cleavage, as previously described (Dodge et al., 2023; Entova et al., 2018). For BCN incorporation, a culture of *E. coli* B.95 cells (B.95.∆A) transformed with the pEvol PylRS AF and pET24 plasmids containing *Cj* PglC amber codon variants was grown as described in the Supporting Methods, Supporting Information S1 (Swiecicki et al., 2020).

PglC variants were purified in SMA200 liponanoparticles as previously described, albeit with a modified washing and labeling procedure on resin to reduce contamination due to nonspecific sticking of SMALPs to the resin and excess dye to SMALPs. The purity of the protein was further improved by size exclusion chromatography to remove excess dye and SMA. Protein concentration was quantified with a Pierce™ bicinchoninic acid protein assay using wildtype SUMO‐PglC as a standard, and fluorophore concentration was determined by Ultraviolet‐visible (UV–vis) to calculate labeling efficiency.

### 4.4 Activity and inhibition assays

The activity of SMALP‐solubilized PglC variants was measured using the UMP‐Glo luminescence assay as previously described, with modifications to the assay buffer for compatibility with liponanoparticles (Figure S4e) (Entova et al., 2018).

Inhibition assays were conducted on detergent‐solubilized SUMO‐*Cj* PglC by measuring activity by UMP‐Glo with or without 100 μM of a given nucleoside analog. For the two highest inhibition compounds, PglC conversion was measured as a function of inhibitor concentration to calculate the half maximal inhibitory concentration (IC_50_)_values. The different compound concentrations were then assayed with a range of substrate concentrations to procure the inhibitory constants (*K*_i_). The compounds were determined to be competitive inhibitors as demonstrated by the changing slopes, in addition to the lines converging upon the *y*‐axis in the Lineweaver–Burk plots (Figure S7).

Additional activity assays were performed using radiolabeled UDP‐sugar substrate to confirm PglC PGT activity is not affected by imaging buffer with the presence of the oxygen scavenging system (Figure S4f) (Arbour et al., 2023).

### 4.5 Functionalized slides

Functionalized glass slides were created with mPEG‐silane‐2000 and either biotin‐mPEG‐silane‐3400 (Laysan Bio) or NTA‐mPEG‐silane‐3400 custom ordered from Nanocs. The glass cleaning and functionalization procedure was modified from the literature protocol to include a chelation step to remove divalent cation contamination (Figure S6) (Gupta et al., 2021).

### 4.6 TIRF microscopy

Fluorescence measurements were performed on an epifluorescence dual‐camera TIRF microscope. DNA mapping oligo controls were ordered from Millipore Sigma and were based on designs from the literature (Gupta et al., 2021; McCann et al., 2010).

Image analysis was performed using custom code written with the MATLAB image processing and curve fitting toolboxes, which are further detailed in the Supporting Methods, Supporting Information S1.

## AUTHOR CONTRIBUTIONS

**Hugh R. Higinbotham:** Conceptualization; investigation; methodology; writing – original draft; validation; visualization; writing – review and editing; formal analysis. **Christine A. Arbour:** Writing – review and editing; methodology; investigation. **Barbara Imperiali:** Conceptualization; writing – original draft; writing – review and editing; funding acquisition; project administration; resources.

## Supporting information


**Data S1.** Detailed procedures for bioinformatic and MD analysis; protein expression and purification, fluorophore labeling, activity assays; slide functionalization and microscope setup; and smFRET data collection and image analysis.

**Video S1.** All‐atom simulations of Chai‐1 prediction of *Cc* PglC in a membrane bilayer for 200 ns. Replicate simulations are aligned by protein with membrane and solvent not displayed.

**Video S2.** All‐atom simulations of Chai‐1 predictions of *Cc* PglC with Mg^2+^, UndP, and UDP‐diNAcBac for 200 ns. Replicate simulations are aligned by protein with membrane and solvent not displayed.

**Video S3.** All‐atom simulations of Chai‐1 predictions of *Ab* ItrA4 in a membrane bilayer for 200 ns. Replicate simulations are aligned by protein with membrane and solvent not displayed.

**Video S4.** All‐atom simulations of Chai‐1 predictions of *Ab* ItrA4 with Mg^2+^, UndP, and UDP‐Gal for 200 ns. Replicate simulations are aligned by protein with membrane and solvent not displayed.

**Video S5.** All‐atom simulations of Chai‐1 predictions of *Bf* WcfS in a membrane bilayer for 200 ns. Replicate simulations are aligned by protein with membrane and solvent not displayed.

**Video S6.** All‐atom simulations of Chai‐1 predictions of *Bf* WcfS with Mg^2+^, UndP, and UDP‐FucNAc4N for 200 ns. Replicate simulations are aligned by protein with membrane and solvent not displayed.

**Video S7.** Extended all‐atom simulation of Chai‐1 prediction of *Cc* PglC with Mg^2+^, UndP, and UDP‐diNAcBac for 1 μs. Membrane bilayer and solvent are not displayed.

## Data Availability

The data that supports the findings of this study are available in the supplementary material of this article.
