# Supplementary material for: Correlating membrane‐protein dynamics with function: Integrating bioinformatics, molecular dynamics, and single‐molecule FRET
Source: Protein Sci. 2025 Oct 23;34(11):e70352. doi: 10.1002/pro.70352 (PMC12550135; doi:10.1002/pro.70352)
Supplement: Supplementary file 1 — Data S1. Detailed procedures for bioinformatic and MD analysis; protein expression and purification, fluorophore labeling, activity assays; slide functionalization and microscope setup; and smFRET data collection and image analysis. [file PRO-34-e70352-s005.docx]

**Supporting Information:**

**Correlating membrane-protein dynamics with function: Integrating bioinformatics, molecular dynamics, and single-molecule FRET**

Hugh Higinbotham^a^, Christine A. Arbour^b^, and Barbara Imperiali^b^*

^a^Department of Biology and Department of Physics, Massachusetts Institute of Technology, Cambridge, MA 02139, USA.

^b^Department of Biology and Department of Chemistry, Massachusetts Institute of Technology, Cambridge, MA 02139, USA.

*Corresponding author: [imper@mit.edu](mailto:imper@mit.edu)

| Hugh R. Higinbotham | 0000-0002-9418-7958 | Dept. of Biology and Dept. of Physics, MIT |
| --- | --- | --- |
| Christine A. Arbour | 0000-0001-6056-296X | Dept. of Biology and Dept. of Chemistry, MIT |
| Barbara Imperiali | 0000-0002-5749-7869 | Dept. of Biology and Dept. of Chemistry, MIT |

**Table of Contents**

**Supplementary Tables**

**Table S1:** SmPGT representative sequences

**Table S2:** Predicted structure RMSDs

**Table S3:** Ligand SMILES

**Table S4:** Bacterial strains

**Table S5:** Mutagenesis primers

**Table S6:** DNA oligomers

**Table S7:** Protein labeling

**Supplementary Figures**

**Figure S1:** Comparison of *Cc* and *Cj* PglC

**Figure S2:** Per-residue RMSF and lDDT analysis for other PGTs

**Figure S3**: Protein stabilization in all-atom MD

**Figure S4:** Protein purification details

**Figure S5:** Functionalized coverslip synthesis and specificity

**Figure S6:** Microscope setup and DNA controls

**Figure S7:** Detailed inhibitor kinetics

**Figure S8:** Full structures and FRET ratio distributions

**Supplementary Methods**

**All-atom MD simulations**

**Structure prediction analysis**

**Site selection and mutagenesis**

**Protein expression of wild-type and single-cysteine variants**

**Non-canonical amino acid mutagenesis**

**Protein purification and fluorophore labeling**

**Activity and inhibition assays**

**Glass coverslip functionalization**

**TIRF Microscopy**

**Microscopy data analysis**

**SUPPLEMENTARY TABLES**

**Table S1:** SmPGT representative sequences

| PGT name | UDP-sugar substrate | Amino acid sequence |
| --- | --- | --- |
| *Cc* PglC | UDP-*N*,*N*'-diacetylbacillosamine | MYRNFLKRVIDILGALFLLILTSPIIIATAIFIYFKVSRDVIFTQARPGLNEKIFKIYKFKTMSDERDANGELLPDDQRLGKFGKLIRSLSLDELPQLFNVLKGDMSFIGPRPLLVEYLPIYNETQKHRHDVRPGITGLAQVNGRNAISWEKKFEYDVYYAKNLSFMLDVKIALQTIEKV  LKRSGVSKEGQATTEKFNGKN |
| *Cj* PglC | UDP-*N*,*N*'-diacetylbacillosamine | MYEKVFKRIFDFILALVLLVLFSPVILITALLLKITQGSVIFTQNRPGLDEKIFKIYKFKTMSDERDEKGELLSDELRLKAFGKIVRSLSLDELLQLFNVLKGDMSFVGPRPLLVEYLPLYNKEQKLRHKVRPGITGWAQVNGRNAISWQKKFELDVYYVKNISFLLDLKIMFLTALKVLKRSGVSKEGHVTTEKFNGKN |
| *Ab* ItrA4 | UDP-Galactose | MIDERKVLPLMNTKLTYPISNEENQFMIIKPQPPLKRVFDLFFAIILFIVLLPLFLLFSIIVKVDGGSAFYGHERVGKNGQKFKCLKFRSMASNSQELLQNLLASDPIAYEEWHSTYKLKNDPRVTKIGHFLRKSSLDEMPQLINIIKGEMSFVGPRPVTQDELTRYKDNVIYYLAVTPGLTGLWQVSGRNDIDYETRVSLDTQYVKNWSFSQDLKILAKTFIIVILGKGAY |
| *Bf* WcfS | 2-acetamido-4-amino-2,4-dideoxy-D-fucose | MSCLIRFFDIVFSLLGILLLSPVFLLLYIAICLESKGGGFYKQLRVGRYGGDFYVYKFRSMRVGADKKGLITVGGRDPRITRTGYLIRKYKLDELPQLFNVLKGDMSLVGPRPEVRKYVDLYTDEQKKVLSVRPGITDYASIEYVDENMILGEASDPDRAYIEQIMPDKIRYNMKYICNRSVKEYFKIIFLTFWSIIR |

**Table S2:** Predicted structure RMSDs

| Chai-1 model rank | Ligands (Mg^2+^, UndP, UDP-diNAcBac) | 8G1N chain A RMSD (Å) | 8G1N chain B RMSD (Å) | AF-A7ZET4 RMSD (Å) | AF-Q0P9D0  RMSD (Å) |
| --- | --- | --- | --- | --- | --- |
| 0 | - | 1.08 | 1.20 | 0.35 | 0.61 |
|  | + | 1.78 | 1.76 | 0.71 | 0.83 |
| 1 | - | 0.92 | 0.94 | 0.57 | 0.73 |
|  | + | 1.74 | 1.74 | 0.71 | 0.82 |
| 2 | - | 2.15 | 2.22 | 0.81 | 0.80 |
|  | + | 1.71 | 1.69 | 0.66 | 0.77 |
| 3 | - | 1.93 | 1.98 | 0.63 | 0.69 |
|  | + | 1.82 | 1.84 | 0.83 | 0.93 |
| 4 | - | 2.01 | 2.10 | 0.73 | 0.73 |
|  | + | 1.75 | 1.79 | 0.72 | 0.83 |
| mean ± σ | - | 1.62 ± 0.51 | 1.69 ± 0.52 | 0.62 ± 0.12 | 0.71 ± 0.06 |
|  | + | 1.76 ± 0.04 | 1.76 ± 0.05 | 0.73 ± 0.06 | 0.84 ± 0.05 |

**Table S3:** Ligand SMILES

| Ligand name | SMILES representation |
| --- | --- |
| Mg^2+^ | [Mg+2] |
| Undecaprenol phosphate | C/C(C)=C/CC/C(C)=C/CC/C(C)=C/CC/C(C)=C/CC/C(C)=C\CC/C(C)=C\CC/C(C)=C\CC/C(C)=C\CC/C(C)=C\CC/C(C)=C\CC/C(C)=C\COP(O)(O)=O |
| UDP-diNAcBac | O[C@@H]([C@H]([C@H](N1C(NC(C=C1)=O)=O)O2)O)[C@H]2COP(OP(O[C@H]3[C@@H](NC(C)=O)[C@H](O)[C@@H](NC(C)=O)[C@H](C)O3)([O-])=O)([O-])=O |
| UDP-FucNAc4N | CC(N[C@H]1[C@@H](OP(OP(OC[C@@]2([H])O[C@@]([H])(N(C=C3)C(NC3=O)=O)[C@@](O)([H])[C@]2([H])O)(O)=O)(O)=O)O[C@H](C)[C@H]([NH3+])[C@@H]1O)=O |
| UDP-Galactose | O=P(OC[C@@H]1[C@H]([C@H]([C@H](N2C(NC(C=C2)=O)=O)O1)O)O)(OP(O[C@@H]3[C@@H]([C@H]([C@H]([C@@H](CO)O3)O)O)O)([O-])=O)[O-] |

**Table S4:** Bacterial strains

| DH5α | Maintained in our lab |
| --- | --- |
| BL21-DE3 RIL | [Lajoie et al., 2013](https://www.sciencedirect.com/science/article/pii/S2451945619303903?via%3Dihub#bib22) (Lajoie et al. 2013) |
| B.95 ΔA | Mukai et al., 2015 (Mukai et al. 2015) |
| C43 pAM174 | [Sjodt et al., 2018](https://elifesciences.org/articles/91125#bib58) (Sjodt et al. 2018) |

**Table S5:** Mutagenesis primers designed with the Agilent QuikChange Primer Design tool. Mutagenesis was confirmed via Sanger sequencing.

| Construct Variant | Primers (Written 5’ to 3’) |
| --- | --- |
| *Cj* PglC-6His E68C | TTTTAAAATTTATAAATTTAAAACCATGAGCGATGAAAGAGAT**TGC**AAGGGTGAGTTATTAAGCGATGA  TCATCGCTTAATAACTCACCCTT**GCA**ATCTCTTTCATCGCTCATGGTTTTAAATTTATAAATTTTAAAA |
| *Cj* PglC-6His E124C | GGTTGAGTATTTGCCTCTTTACAATAAA**TGC**CAAAAATTGCGTCATAAAGTGCGTCC  GGACGCACTTTATGACGCAATTTTTG**GCA**TTTATTGTAAAGAGGCAAATACTCAACC |
| *Cj* PglC-6His L166TAG | AAAATTCGAACTTGATGTGTATTATGTGAAAAATATTTCTTTT**TAG**CTTGATTTAAAAATCATGTTTTTAACAG  CTGTTAAAAACATGATTTTTAAATCAAG**CTA**AAAAGAAATATTTTTCACATAATACACATCAAGTTCGAATTTT |
| *Cj* PglC-SII E68C | AACCATGTCCGACGAGCGCGAT**TGC**AAGGGCGAATTGC  GCAATTCGCCCTT**GCA**ATCGCGCTCGTCGGACATGGTT |
| *Cj* PglC-SII E124C | 5’ GGTGGAATACCTGCCGCTGTACAATAAA**TGC**CAGAAGCTGCGCCAC  5’ GTGGCGCAGCTTCTG**GCA**TTTATTGTACAGCGGCAGGTATTCCACC |

**Table S6:** Functionalized DNA oligomers for imaging controls

| Name | Sequence | Vendor |
| --- | --- | --- |
| Mapping – biotin | 5’−/Bio/CCAAAGACACCACAGACCACACACAAGACAACGTCGTGACTGGGAAAACCCT−3’ | Millipore Sigma |
| Mapping – Alexa488 | 5’−/Alexa488/AGGGTTTTCCCAGTCACGAC-3′ | Millipore Sigma |
| Mapping – Cy3 | 5’−GTGTCCCTCTCGAT/Cy3/−3′ | Millipore Sigma |
| Mapping – Cy5 | 5’−GTGTGTGGTCTGTGGTGTCT/Cy5/−3′ | Millipore Sigma |
| FRET – Cy3, Biotin | /5Cy3/GG ACT GCC GCC TGC GGA GCC GCA CGA CGA CAC GAC AAA G/3Bio/ | Millipore Sigma |
| FRET – Cy5 | C GTGT CGTC GTGC GGCT CCGC AGGC G/3Cy5sp/ | Millipore Sigma |

**Table S7:** Protein labeling quantification.

| PglC variant | [Protein] ± 0.5 μM | [Cy3] | [Cy5] | % Cy3 | % Cy5 |
| --- | --- | --- | --- | --- | --- |
| E68C | 7.5 μM | - | 3.55 μM | - | ~50% |
| E124C | 7.8 μM | 3.23 μM | - | ~40% | - |
| E68C-L166BCN | 1.5 μM | 0.79 μM | 0.99 μM | ~60% | ~70% |
| E124C-L166BCN | 3.1 μM | 1.47 μM | 1.12 μM | ~50% | ~40% |

**
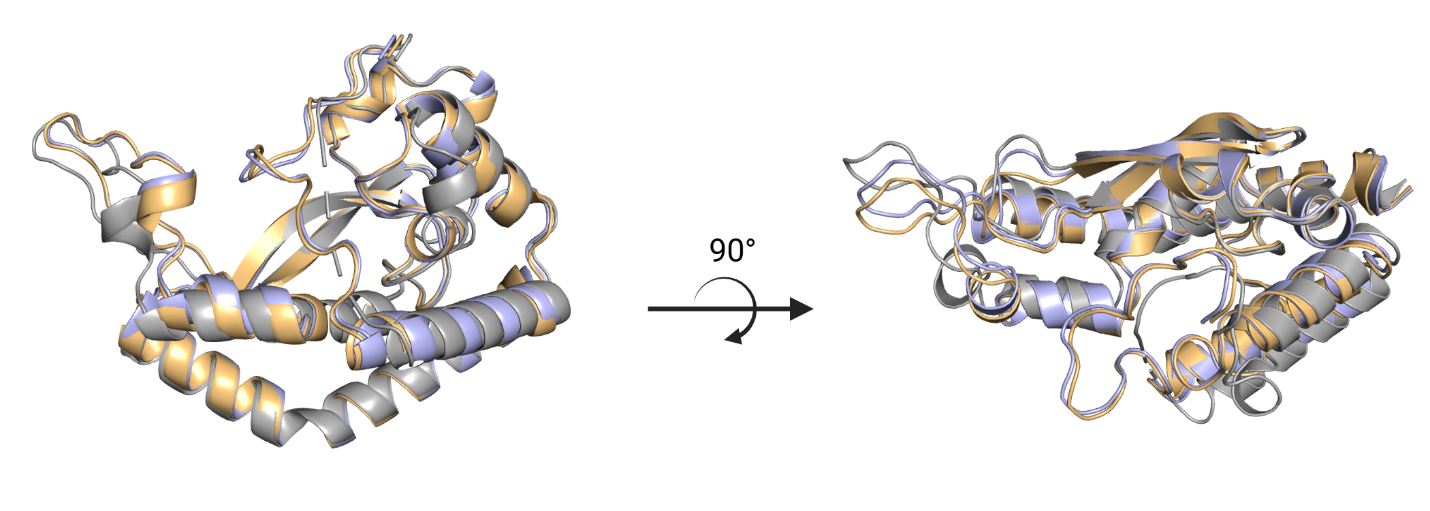
SUPPLEMENTARY FIGURES**

**Figure S1: Comparison of Cc and Cj PglC**. A superposition of cartoon representations of the Cc PglC crystal structure chain A from PDB ID: 8G1N (gray), the AlphaFold2 prediction of Cc PglC AF-A7ZET5-F1 (blue), and the AlphaFold2 prediction of Cj PglC AF-Q0P9D0-F1 (orange) is shown. Both AlphaFold2 predictions have < 1.44 Å RMSD of the crystal structure, and the predicted structures of the two strains have RMSD = 0.47 Å from each other. Cc and Cj PglC have 71.64% sequence identity (144 identical residues out of 201 aligned positions) and 82.09% similarity (21 additional aligned residues are chemically similar amino acids).

**
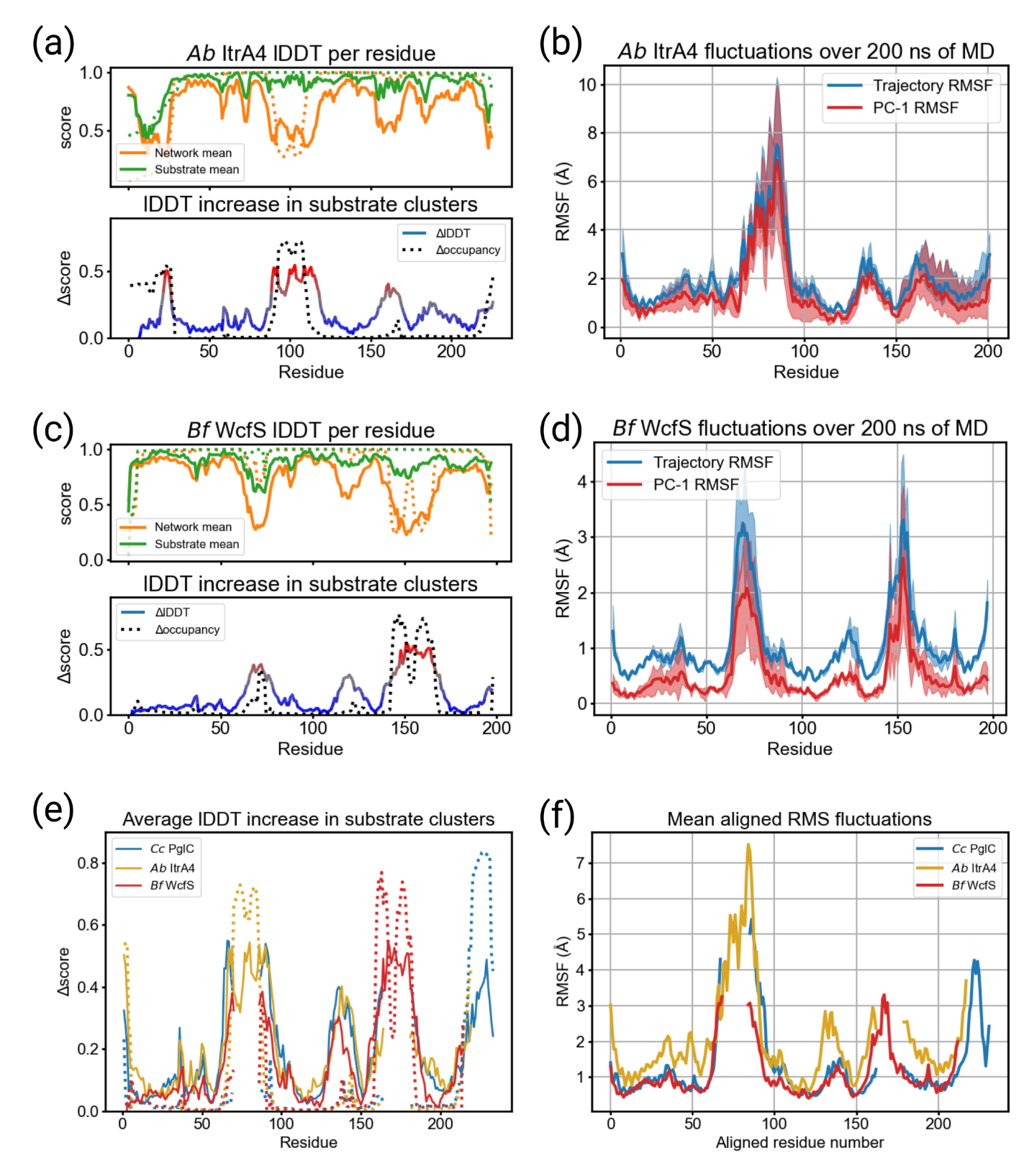
**

**Figure S2: Per-residue RMSF and lDDT analysis for other PGTs.** (A), (C): Top: lDDT per residue against all SmPGTs (orange) and against all members of the same substrate-specific cluster (green). Solid lines indicate average lDDT against residues aligned to the same position and dashed lines indicate fractional occupancy of aligned residue at that site. (B), (D): All fluctuations (blue) and PC-1 fluctuations (red). Solid line is the average of three replicates with shading to show the range of values. (E): Difference between network and cluster lDDT aligned by sequence. The same analogous and dynamic structural motifs are highly substrate specific. Relative occupancy only highlights substrate-specific regions of variable length. (F): Average RMS fluctuations for all three PGTs when aligned to each other by amino acid sequence. Analogous regions of each protein show elevated dynamics.

**
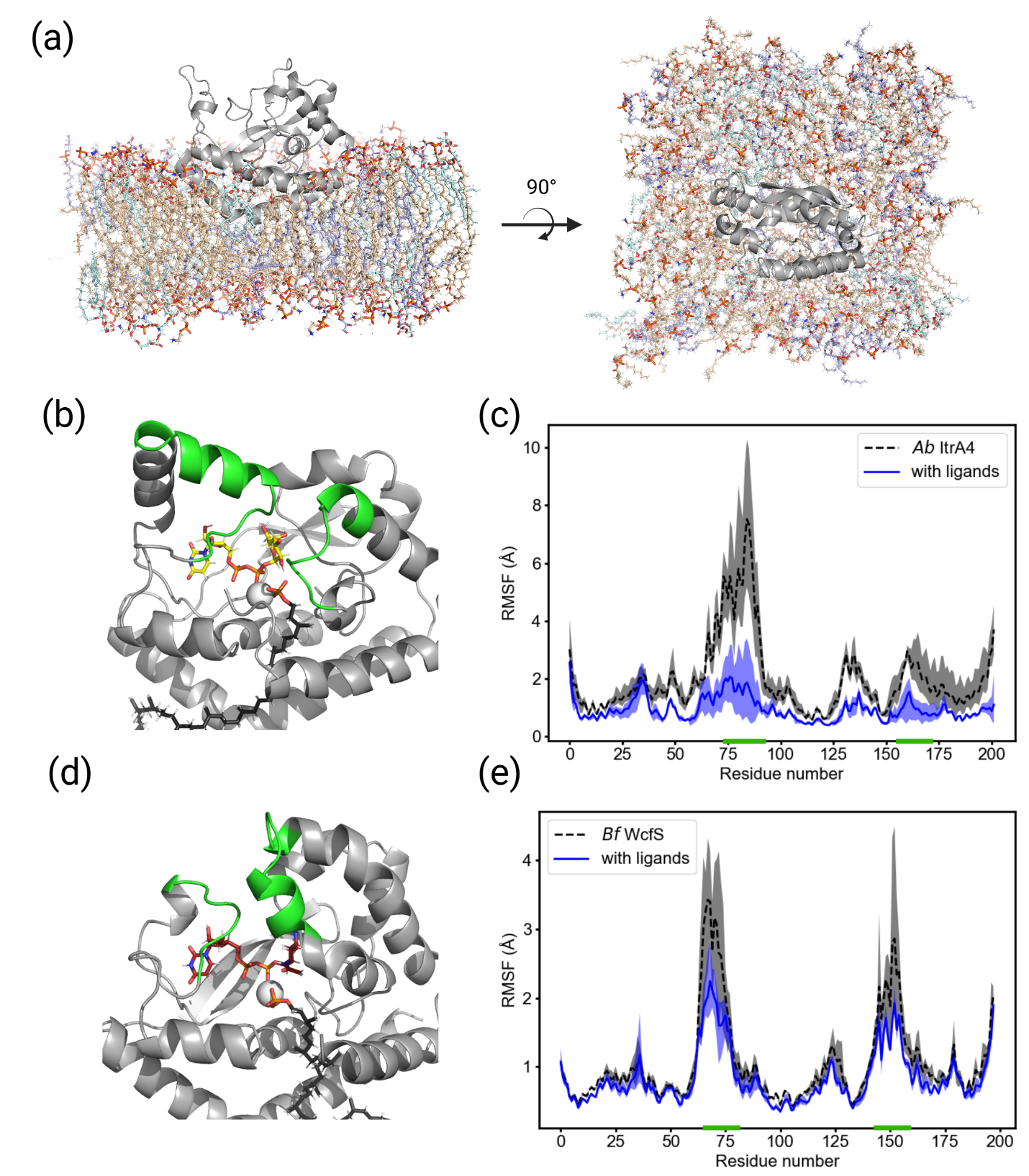
**

**Figure S3: Protein stabilization in all-atom MD.** (A): Snapshot of C. concisus PglC equilibrated in membrane. Tan is POPE, blue is POPG, and cyan is Cardiolipin. (B): Snapshot of A. baumannii. ItrA4 with UDP-galactose (yellow) and UndP (black) after 200 ns of simulation in a phospholipid bilayer. Green highlighted regions showed the most dramatic stabilization and correspond to substrate-specific structures. (C): Average RMSF per residue over three replicates of 200 ns simulations with or without ligands. Green highlighted regions shown on the x-axis. (D): B. fragilis. WcfS snapshot with UDP-FucNAc4N (red) and UndP (black) after 200 ns simulation. (E) WcfS fluctuations per residue with or without ligands present over three replicate simulations.


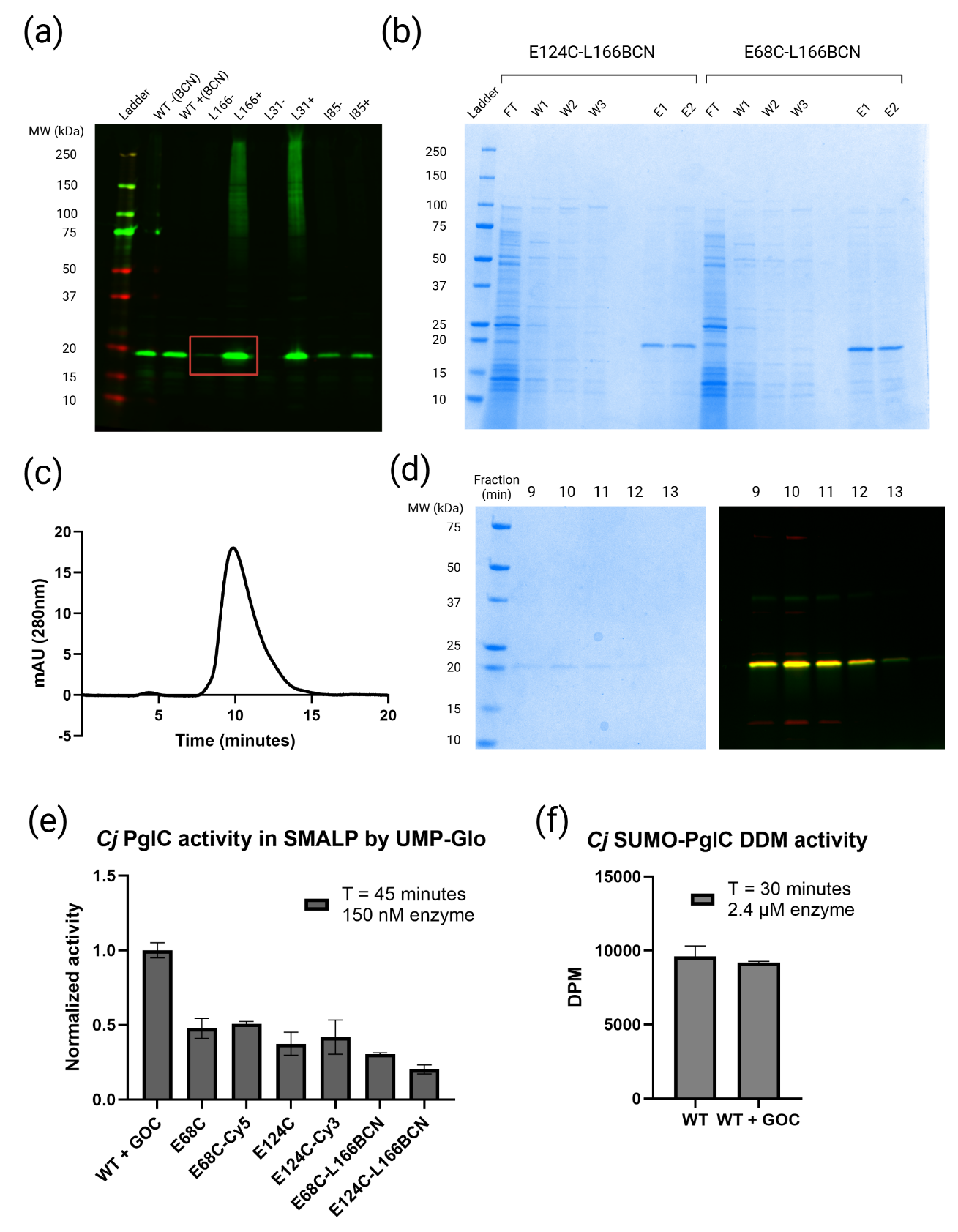


**Figure S4: Purification and characterization details for Cj. PglC dual-labeled variants.** (A): Fluorescence western of test cultures of B95 cells expressing BCN point variants of Cj.PglC. L166 showed strong suppression without BCN and the best expression with BCN present. (B). Coomassie gel of initial Ni-NTA purification of BCN, cysteine dual variants. (C): UV absorption trace from size exclusion column on loop-labeled variant E68C-L166BCN. 0.5 ml fractions were collected every 30 seconds. (D) Left: Coomassie gel of every-other SEC fraction, labeled by minute collected. Right: Fluorescence composite of SEC fractions taken prior to Coomassie staining. Fractions were combined for the fluorescence gel in Figure 3. (E): Activity of Cj PglC variants in the UMP-Glo assay. (F): Radioactivity-based assay of detergent-solubilized SUMO-PglC. Oxygen scavenging reaction does not significantly affect activity.


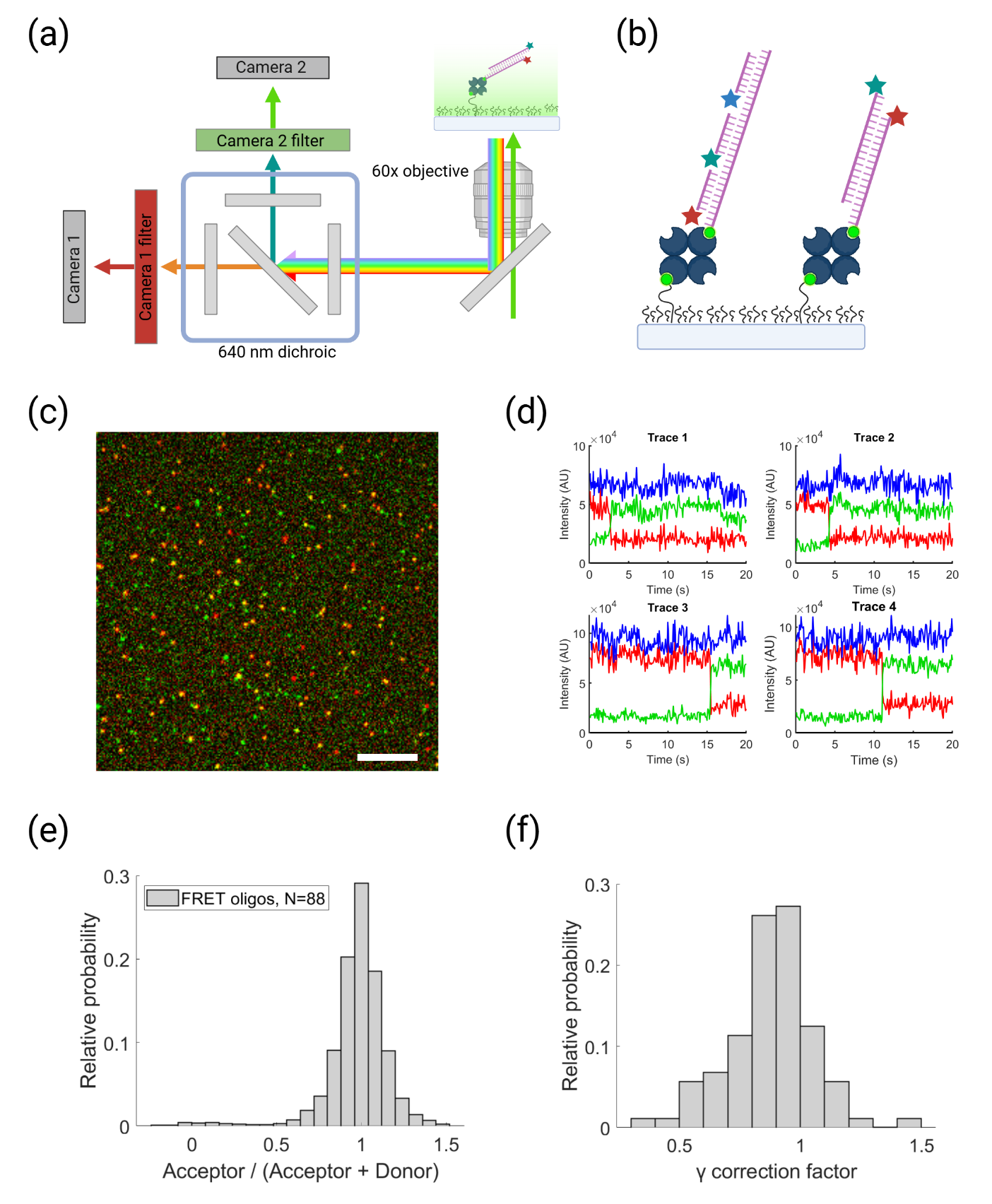


**Figure S5: Microscope setup and DNA-oligo controls.** (A): Microscope diagram illustrating dual camera dichroic setup. (B): Schematic of biotin-streptavidin slides and mapping oligomers (left) versus FRET oligomers (right). (C) Overlay of Cy3 and Cy5 from mapping oligomers illustrating manual camera registration. One pixel tolerance sufficed to correctly map colocalized points across the field of view. (D) Sample FRET traces illustrating γ factor near 1 after manual adjusting gain between cameras. Green is Cy3 emission, red is Cy5 emission, and blue is total intensity that will remain constant for a γ factor of 1. (E): FRET ratio distribution for DNA FRET oligomers. (F): Corresponding γ distribution for the same FRET traces of these oligomers. For subsequent experiments γ outside of [0.75,1.25] was used to filter lower quality traces.


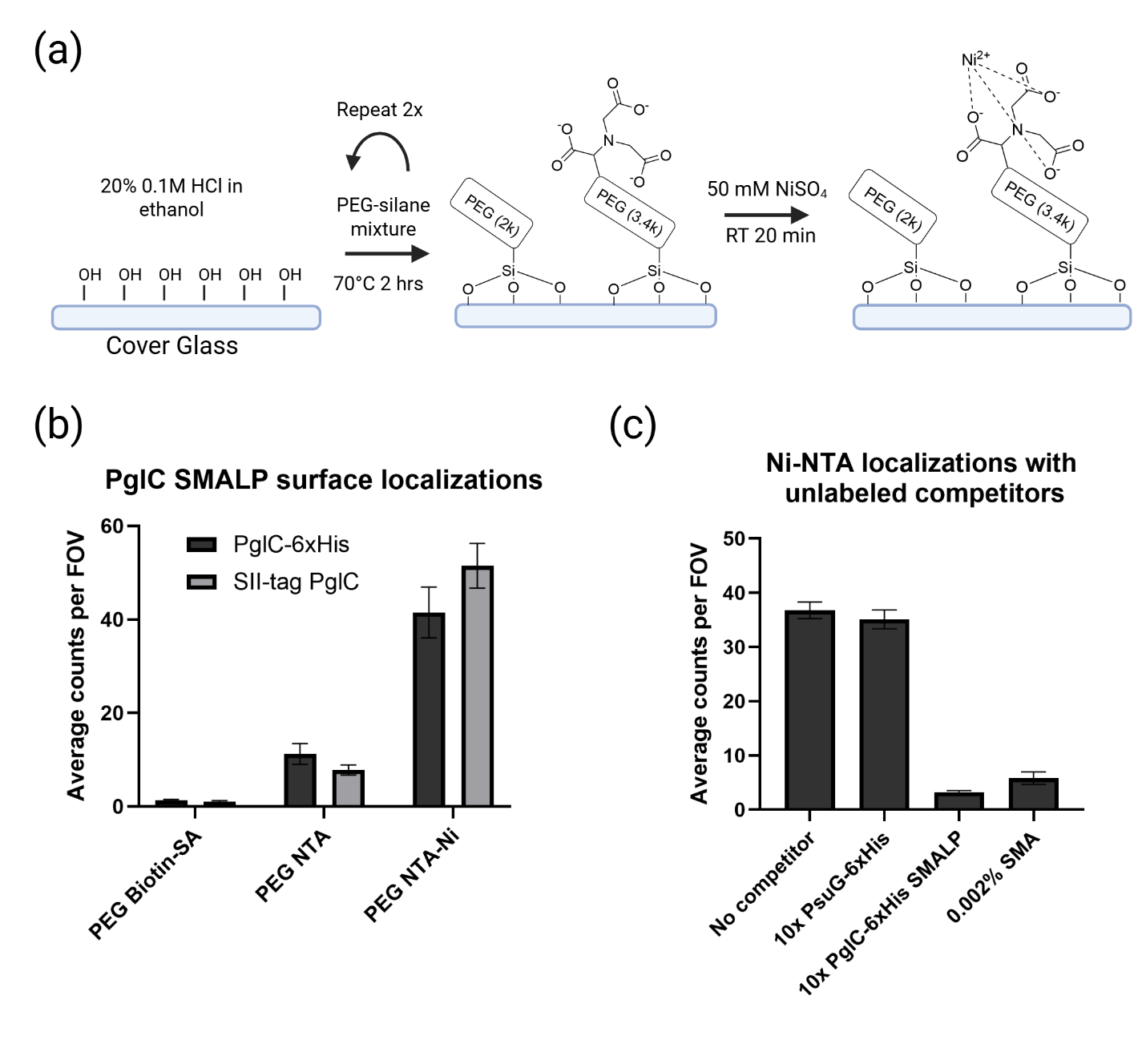


**Figure S6: Slide synthesis and specificity controls.** (A) Surface functionalization scheme for labeling protein. (B) Number of single-molecule localizations (determined by single-step photobleaching) per field of view for different slide surface chemistries, 50:1 mass ratio of PEG to functionalized-PEG. Similar behavior observed for SMALPs purified with 6xHis and SII tags. (C) Competition experiments to test for specificity of surface binding. Unlabeled SMALPs or free SMA compete with labeled SMALPs for surface binding sites, while soluble protein with a 6xHis tag does not. All error bars are given as SEM across 10 – 20 separate fields of view (FOV).

**
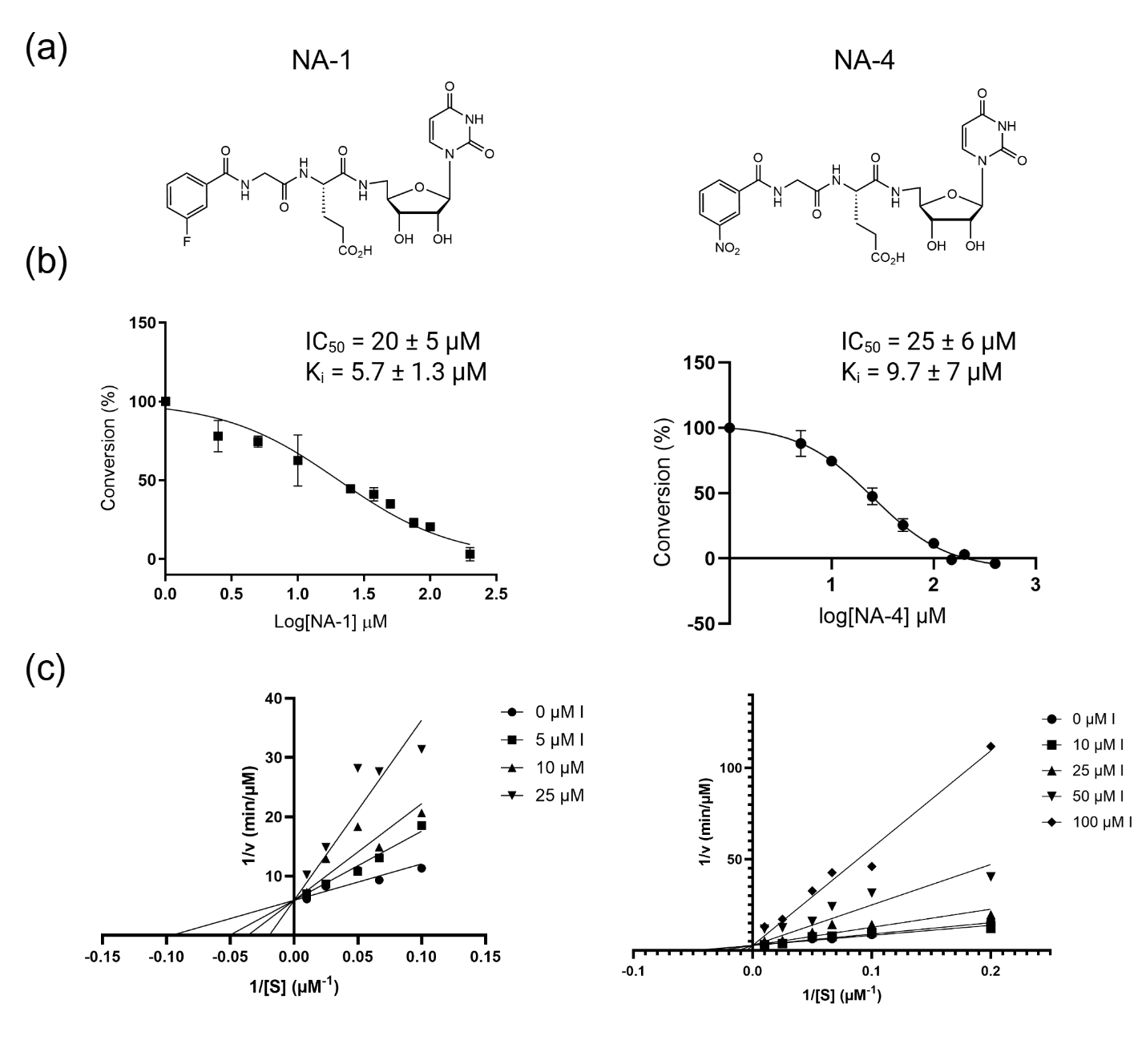
**

**Figure S7: Kinetic analysis of strongest nucleoside inhibitors NA-1 and NA-4.** (A): Chemical structures of nucleoside analog inhibitors. (B): IC_50_ curves of select inhibitors with C. jejuni PglC, measured by UMP-Glo monitoring luminescence. Percent conversion was determined after pre-incubation with inhibitor as described by the UMP-Glo Assay procedure, in reference to control with no inhibitor. Error bars indicate mean and error ± SD; n = 2. (C): Lineweaver-Burk plot of C. jejuni PglC with compounds NA-1 and NA-4 and UDP-diNAcBac. The substrate conversion was monitored by UMP-Glo luminescence assay.

**
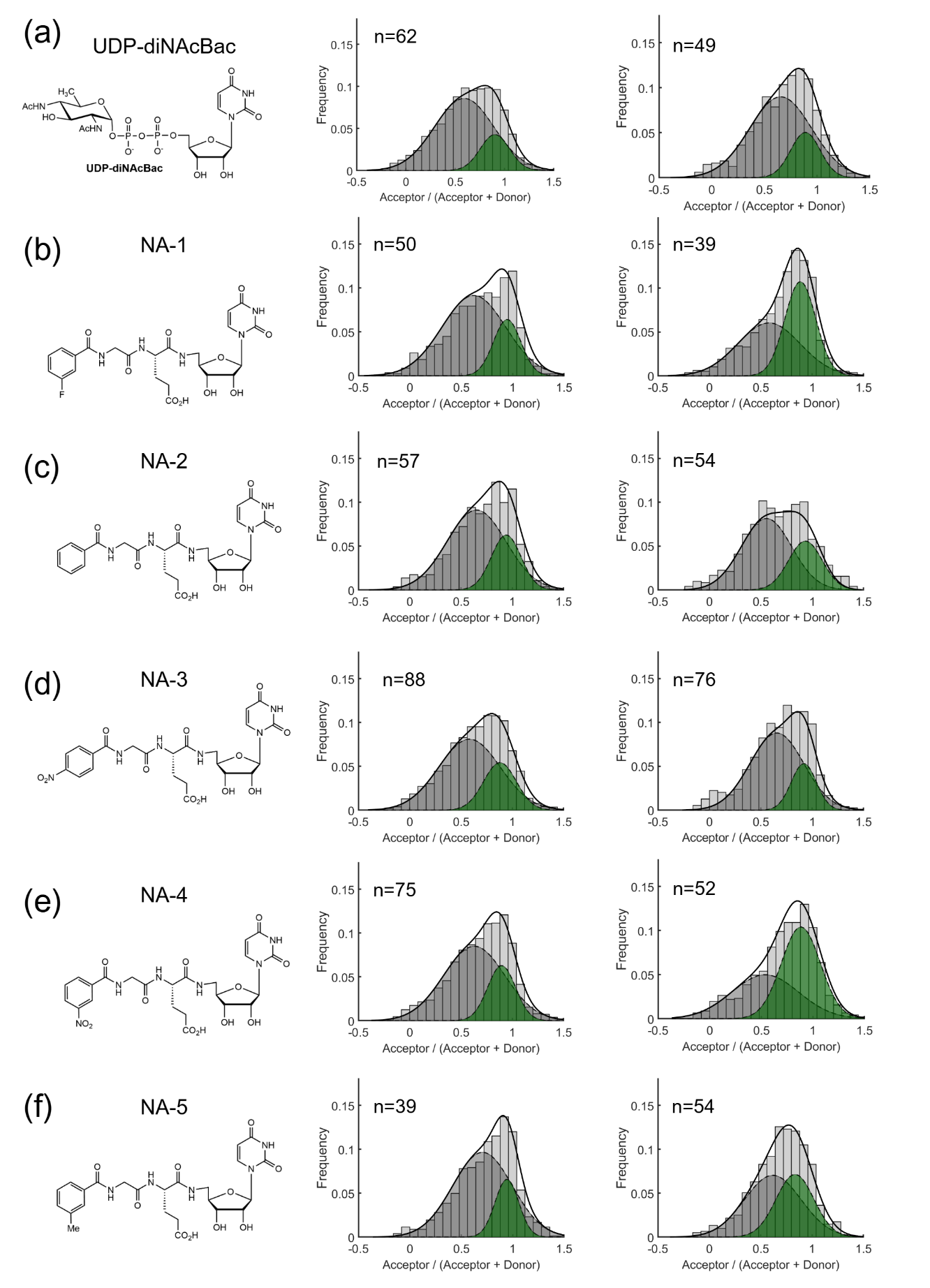
**

**Figure S8: FRET ratio distributions for full ligand screen.** (A)-(F): Left: Full chemical structure of UDP-diNAcBac or nucleoside analog. Middle: Control distribution and fits taken for each inhibitor without ligand present. Right: Distribution and fits of FRET ratios in the presence of each ligand.

**SUPPLEMENTARY METHODS**

**All-atom MD simulations**

All proteins were simulated in a lipid bilayer composed of 67 mol% 1-palmitoyl-2-oleoyl-sn-glycero-3-phosphoethanolamine (POPE), 23 mol% 1-palmitoyl-2-oleoyl-sn-glycero-3-phosphoglycerol (POPG), and 10 mol% cardiolipin (CL) of defined acyl-chain composition using the CHARMM36 all-atom force field (MacKerell et al. 1998). Lipid bilayer systems were solvated using the TIP3P water model with 0.15 M KCl and run at a temperature of 303 K. The CHARMM general Force-Field (CgenFF) webserver was used to generate topologies for UndP and UDP-diNAcBac (Vanommeslaeghe et al. 2010).

Each system was equilibrated for at least 50 ns following the CHARMM-GUI protocol with additional CHARMM relaxation and inputs generated for GROMACS (Wu et al. 2014). All simulations were performed using the GROMACS program version 2024.1 unless otherwise stated.

Principal component analysis, residue contacts, and root-mean square fluctuations were computed using the MDAnalysis library in python 3.12.

**Structure prediction analysis**

The three representative PGTs were chosen had distinct UDP-sugar substrate selectivity as confirmed via UMP-Glo (Durand et al. 2024). Additionally, *Cc.* PglC is the only SmPGT with a solved structure at time of writing, and *Ab.* ItrA4 is a close structural and sequence analog of the catalytic domain of *Se.* WbaP which has been solved by Cryo-EM (Dodge et al. 2024).

We created a local Foldseek database of the AlphaFold 2 predictions of the entire SmPGT network and queried each representative using Foldseek easy-search with flags –alignment-type 1, --exhaustive-search, --format-output “query target alntmscore lddtfull”, which performed structural alignments using the accelerated TMalign for a global alignment. The lDDT and occupancy was computed per residue from the full output by averaging over the appropriate subset of PGTs for a given cluster/network comparison.

**Protein expression of wild-type and single-cysteine variants**

Mutagenesis sites were selected at positions with low amino acid conservation scores when aligned to the family of small PGTs in the SmPGT SSN. (Durand et al. 2024) PglC from *C. jejuni* was mutated using QuikChange to replace specific residues with cysteines or the amber stop codon TAG. Primers were designed with the QuikChange online primer tool (Agilent) and successful mutations were confirmed via Sanger sequencing (Table S2).

Wild-type (WT) and SUMO-WT protein with C-terminal 6xHis purification tag were overexpressed using the Studier autoinduction method in the *E. coli* strain Bl21 (Studier 2005)

For Strep-tagged variants, C43 cells harboring pAM174 (Sjodt et al. 2018) were transformed with a plasmid encoding *C. jejuni* PglC with an N-terminal SUMO tag – linker – dual-strep tag sequence (Dodge et al. 2023). Cells were also expressed by autoinduction with the addition of 1 g solid (L)-arabinose at OD_600_ ~1.5 to induce SUMO cleavage before the temperature was reduced to 17 °C. Cells were harvested by centrifugation, flash frozen, and stored at -80°C. (Swiecicki et al. 2020)

**Non-canonical amino acid mutagenesis**

BCN variants were grown in 0.5ml test cultures (see below) and assessed by anti-His western blot for expression in the presence of BCN and suppression in its absence to ensure proper ncAA incorporation (Figure S3A).

A culture of *E. coli* B.95 cells (B.95.∆A) transformed with the pEvol PylRS AF and pET24 plasmids containing PglC amber codon variants were incubated overnight in LB containing chloramphenicol and kanamycin (200 rpm; 37 °C). 100 mL of an LB broth shaking culture (200 rpm; 37 °C) supplemented with chloramphenicol and ampicillin for selection were inoculated with 250 µL of the overnight culture (dilution x200). A stock of 80 mM BCN unnatural amino acid in 0.2 M NaOH, 15% DMSO, was prepared. At an OD of 0.2, the stock solution of BCN unnatural amino acid was diluted down to 16 mM in 1 M HEPES buffer, pH 7.5, and finally added to final concentration of 1 mM. Simultaneously, cells were induced with 0.05 % L-arabinose (20 % stock in water). The culture was further incubated for 40 minutes at 37 ° C. Cells were finally induced with IPTG (final concentration of 0.2 mM) and incubated at 25 °C overnight. Cells were harvested by centrifugation, flash frozen, and stored at –80 °C (Swiecicki et al. 2020).

**Protein purification and fluorophore labeling**

WT-SUMO *Cj* PglC was purified in n-Dodecyl-β-D-Maltoside (DDM) as previously described (Anderson et al. 2023).

Protein was purified in SMALP200 liponanoparticles as described in (Dodge et al. 2023). After Ni-NTA or Strep-Tactin XT 4flow resin pulldown, protein was desalted with Zeba spin desalting columns into SMALP buffer (150 mM NaCl with 50 mM HEPES at pH 8.0). Protein concentration was quantified with a Pierce™ BCA protein assay kits and incubated for 2 hours at room temperature with a 10x molar excess of sulfo-Cy3 or sulfo-Cy5 maleimide dye (Lumiprobe). For dual labeled variants, an additional labeling step was conducted for 2 hours at room temperature with Cy3-tetrazine dye.

To remove excess dye and SMA, we purified the sample using size exclusion chromatography as a final step (Figure S3C-D) (Dodge et al. 2024).

**Activity and Inhibition Assays**

The activity of SMALP solubilized proteins was confirmed using the UMP-Glo luminescence assay as previously described (Entova et al. 2018). Given the reduced turnover of UndP in SMALPs, enzyme was pre-incubated with UndP for 30 minutes and a higher concentration was used relative to previously reported assays. Assays were incubated and in SMALP buffer with 1 mM MgCl_2_, 10% DMSO, 100 μM UndP, and the reaction was initiated with the addition of 20 μM UDP-diNAcBac.

Inhibition assays were conducted using UMP-Glo [Promega cat. VA1130] as previously described (Durand et al. 2024), which detects UMP release from the phosphoglycosyl transferase reactions. Due to the structural similarity between the synthesized inhibitors and UMP, we also controlled for off-target inhibition of the Glo reagent enzymes by the nucleoside analogues. To correct for off-target inhibition, 1.5 µM UMP (the amount typically released during the PglC reactions) was combined with inhibitor or DMSO (control) followed by the addition of the detection reagent. The percentage of UMP signal lost was used to adjust the luminescence readout (Seebald et al. 2020). The quenching solution was prepared as described by Promega. Assays contained 10% DMSO, 0.1% Triton X-100, 0.05 mg/mL (0.76 µM) Bovine Serum Albumin (BSA), 50 mM HEPES at pH 7.5, 100 mM NaCl, 5 mM MgCl_2_, 20 μM UndP (from either 10X stock: 200 µM or 20X stock: 400 µM in DMSO), 20 μM UDP-diNAcBac (from 10X stock: 200 µM in Milli-Q H_2_O), and 0.5 nM *C. jejuni* PglC (from 10X stock: 5 nM in *Cj* PglC buffer) in a final volume of 12 μL. Inhibitors were added from a 2 mM stock in DMSO, at a final concentration of 100 μM. PglC was preincubated in the reaction mixture lacking UDP-diNAcBac for 10 min at ambient temperature. The reaction rate was predetermined to be linear over 15 min at the given concentrations. Upon the addition of UDP-diNAcBac, the reaction was allowed to proceed for 9 min before the addition of quenching solution (12 µL). The reaction mixture (20 µL) was transferred to a 96-well plate (white, nonbinding surface, Corning). The plate was shaken at low speed for 16 min and incubated for 44 min at ambient temperature, and luminescence was read using a Synergy H1 hybrid plate reader (Biotek). Data were plotted using GraphPad Prism, as percentage inhibition compared to the positive control (no inhibitor). PglC buffer for enzyme dilutions: 50 mM HEPES at pH 7.5, 100 mM NaCl, 5 mM MgCl_2_ with 0.2% DDM.

To establish the competitive mechanism of action of the inhibitors, the compounds were screened at varying concentrations with a range of substrate concentrations (UDP-diNAcBac 10, 15, 20, 40, and 100 µM for compound NA-1 and 5, 10, 15, 20, 40, and 100 µM for NA-4). Assays in the absence of inhibitors were performed to establish linear kinetics for *Cj* PglC (0.5 nM) with the range of substrate concentrations using modified conditions described above. The assays were then performed in the presence of inhibitors and were quenched within the linear range (6-10 min depending on substrate concentration). The reciprocal of the initial reaction velocity (1/v) was plotted against the reciprocal of the substrate concentration (1/[UDP-diNAcBac]). Both inhibitors were determined to be competitive due to the converging lines on the y-axis in the Lineweaver-Burk plots.

Additional activity assays were performed using tritiated UDP-sugar substrate to monitor product formation (Arbour et al. 2023) in detergent solubilized SUMO-PglC with or without the presence of the oxygen scavenging system. Activity is reported as the disintegrations per minute (DPM) in the organic layer, with error bars given by standard deviation for n = 2 replicates.

**Glass coverslip functionalization**

Functionalized glass coverslips were created as described with biotin-mPEG-silane-3400 (Laysan Bio) or NTA-mPEG-silane-3400 custom ordered from Nanocs (Gupta et al. 2021).

Coverslips were incubated on the day of the experiment with 100 mM EDTA pH 7.5 for 15 minutes to remove divalent cation contamination before washing with 10 mM HEPES pH 8. This was found to reduce non-specific tethering of SMALPs to both biotinylated or Ni-NTA slides (Figure S3B). Biotin slides were incubated with a 400x dilution of 5 mg/mL streptavidin aliquots for 1 minute before biotinylated DNA constructs were flown in and incubated for an additional 1 minute. NTA slides were incubated with 100 mM EDTA pH 8 for 15 minutes to remove divalent cation contamination, and prior to imaging, slides were then washed with 10 mM HEPES pH 8 and incubated with 50 mM NiSO_4_ for 30 minutes.

**TIRF Microscopy**

Fluorescence measurements were performed on an epifluorescence TIRF microscope. The setup included an Eclipse Ti microscope (Nikon) equipped with a 60× Apo-TIRF oil immersion objective lens (NA 1.49; Nikon) placed on a vibration cancellation table (TMC). Labeled proteins in the evanescent field were excited at 532 nm and fluorescence was imaged on a dual-camera ImagEM EM-CCD setup (Hamamatsu), where acceptor and donor emission were separated through a dichroic beam splitter.

DNA mapping oligo controls (Friedman et al. 2006) were used to manually register the two cameras, while DNA oligomers designed for high FRET transfer were used to adjust camera gain for a gamma factor approximately equal to 1 between the two color channels (Figure S5A).

The imaging buffer consists of 0.2 μm filtered SMALP buffer with 5 mg/mL bovine serum albumin and 0.05% tween 20 detergent to reduce non-specific interactions with the coverslip.

The oxygen scavenging system consisted of glucose oxidase and bovine catalase (GOC) with Trolox to reduce triplet-state quenching (Gupta et al. 2021). On the day of the experiment lyophilized aliquots of GOC were resuspended to a 10X stock solution and syringe filtered through a 0.2 μm PES membrane. Immediately prior to imaging 100 μL of oxygen scavenging buffer was mixed to a final concentration of 1X GOC (40 U/mL glucose oxidase, 1500 U/mL catalase), 1 mM MgCl_2_, 2 mM Trolox, 2% DMSO, 50 mM D-glucose, and 100 μM inhibitor. Trolox was diluted 100X from a pure DMSO stock, and inhibitors were diluted 10X from a 10% DMSO stock.

Protein SMALP samples were diluted to ~10 nM in imaging buffer. Flow chambers on functionalized slides were wetted with the same buffer before labeled protein was perfused in and incubated for 30-minutes at room temperature on the slide. After washing with 200 μL of imaging buffer to remove untethered protein, the oxygen scavenging system was mixed and perfused over the slide immediately prior to imaging. Oxygen scavenger was replenished every 30 minutes to prevent buffer acidification.

**Microscopy data analysis**

Raw TIFF image stacks were background subtracted using a top-hat filter with a 4-pixel radius disk as a structuring element. Relative intensities still vary over the field of view with laser intensity, so a flatfield image was taken using free dye and used to correct for local intensity.

Point particles were picked from maximum intensity projections for each color channel using the Crocker and Grier algorithm and fit to 2D Gaussians to be filtered by size and intensity (Crocker and Grier 1996; Friedman et al. 2006). Total intensity was integrated in a 2x2 pixel square region around particles sufficiently far from neighbors and local background was estimated from adjacent pixels and averaged over the duration of each video.

Slide specificity was measured by counting Cy5 surface localizations that photobleached in a single step per field of view (FOV) (Figure S4B). Competition experiments featured an additional 10X concentration of unlabeled competitor for surface binding sites (Figure S4C).

For FRET imaging, frames were alternated between 640nm and 532nm illumination to monitor the presence of both fluorophores. Traces were kept that showed simultaneous donor recovery upon acceptor bleaching and an observed γ factor of 1 ± 0.2. These were then filtered by intensity Cy5 emission intensity with 640nm excitation and Cy3 emission intensity upon 532 nm excitation after Cy5 photobleaching. Thresholds were set from the singly labeled protein-Cy3 and protein-Cy5 distributions to ensure single molecules of Cy3 and Cy5. The FRET ratio was calculated as the ratio of the acceptor emission to the total emission intensity for the trace prior to acceptor photobleaching. Traces with 10 or more time points exhibiting FRET were added to the overall distribution for a given condition. Empirical FRET was calculated as the ratio of Cy5 to total emission under 532 nm illumination.

For each FRET distribution, the Kernel Density Estimation (KDE) was calculated fit to the sum of two Gaussians with fitting parameters restricted to [0,∞) using the MATLAB curve fitting toolbox. Gaussian fits to Kernel Density Estimation of eFRET distributions were calculated using the MATLAB curve fitting toolbox to the function:

$f\left( x \right)=a_{1}\exp\left( -\left( \frac{x-b_{1}}{c_{1}} \right)^{2} \right)+a_{2}exp\left( -\left( \frac{x-b_{2}}{c_{2}} \right)^{2} \right)$

The relative area of the two Gaussians were calculated from the fitted values and the uncertainty was set by the minimum and maximum relative area within the 95% confidence interval of the fitting parameters.

**References**

1. Anderson AJ, Dodge GJ, Allen KN, Imperiali B (2023) Co-conserved sequence motifs are predictive of substrate specificity in a family of monotopic phosphoglycosyl transferases. Protein Sci 32:e4646.

2. Arbour CA, Nagar R, Bernstein HM, Ghosh S, Al-Sammarraie Y, Dorfmueller HC, Ferguson MAJ, Stanley-Wall NR, Imperiali B (2023) Defining early steps in Bacillus subtilis biofilm biosynthesis. mBio 14:e00948-00923.

3. Crocker JC, Grier DG (1996) Methods of Digital Video Microscopy for Colloidal Studies. J Colloid Interface Sci 179:298-310.

4. Dodge GJ, Anderson AJ, He Y, Liu W, Viner R, Imperiali B (2024) Mapping the architecture of the initiating phosphoglycosyl transferase from S. enterica O-antigen biosynthesis in a liponanoparticle. eLife 12:RP91125.

5. Dodge GJ, Bernstein HM, Imperiali B (2023) A generalizable protocol for expression and purification of membrane-bound bacterial phosphoglycosyl transferases in liponanoparticles. Protein Expression Purif 207:106273.

6. Durand T, Dodge GJ, Siuda RP, Higinbotham HR, Arbour CA, Ghosh S, Allen KN, Imperiali B (2024) Proteome-wide bioinformatic annotation and functional validation of the monotopic phosphoglycosyl transferase superfamily. Proc Natl Acad Sci USA 121:e2417572121.

7. Entova S, Billod J-M, Swiecicki J-M, Martín-Santamaría S, Imperiali B (2018) Insights into the key determinants of membrane protein topology enable the identification of new monotopic folds. eLife 7:e40889.

8. Friedman LJ, Chung J, Gelles J (2006) Viewing Dynamic Assembly of Molecular Complexes by Multi-Wavelength Single-Molecule Fluorescence. Biophys J 91:1023-1031.

9. Gupta S, Friedman LJ, Gelles J, Bell SP (2021) A helicase-tethered ORC flip enables bidirectional helicase loading. eLife 10:e74282.

10. Lajoie MJ, Rovner AJ, Goodman DB, Aerni H-R, Haimovich AD, Kuznetsov G, Mercer JA, Wang HH, Carr PA, Mosberg JA, Rohland N, Schultz PG, Jacobson JM, Rinehart J, Church GM, Isaacs FJ (2013) Genomically Recoded Organisms Expand Biological Functions. Science 342:357-360.

11. MacKerell AD, Bashford D, Bellott M, Dunbrack RL, Evanseck JD, Field MJ, Fischer S, Gao J, Guo H, Ha S, Joseph-McCarthy D, Kuchnir L, Kuczera K, Lau FTK, Mattos C, Michnick S, Ngo T, Nguyen DT, Prodhom B, Reiher WE, Roux B, Schlenkrich M, Smith JC, Stote R, Straub J, Watanabe M, Wiórkiewicz-Kuczera J, Yin D, Karplus M (1998) All-Atom Empirical Potential for Molecular Modeling and Dynamics Studies of Proteins. J Phys Chem B 102:3586-3616.

12. Mukai T, Hoshi H, Ohtake K, Takahashi M, Yamaguchi A, Hayashi A, Yokoyama S, Sakamoto K (2015) Highly reproductive Escherichia coli cells with no specific assignment to the UAG codon. Sci Rep 5:9699.

13. Seebald L, Madec AGE, Imperiali B (2020) Deploying Fluorescent Nucleoside Analogues for High-Throughput Inhibitor Screening. ChemBioChem 21:108-112.

14. Sjodt M, Brock K, Dobihal G, Rohs PDA, Green AG, Hopf TA, Meeske AJ, Srisuknimit V, Kahne D, Walker S, Marks DS, Bernhardt TG, Rudner DZ, Kruse AC (2018) Structure of the peptidoglycan polymerase RodA resolved by evolutionary coupling analysis. Nature 556:118-121.

15. Studier FW (2005) Protein production by auto-induction in high-density shaking cultures. Protein Expression Purif 41:207-234.

16. Swiecicki JM, Santana JT, Imperiali B (2020) A Strategic Approach for Fluorescence Imaging of Membrane Proteins in a Native-like Environment. Cell Chem Biol 27:245-251.e243.

17. Vanommeslaeghe K, Hatcher E, Acharya C, Kundu S, Zhong S, Shim J, Darian E, Guvench O, Lopes P, Vorobyov I, Mackerell Jr. AD (2010) CHARMM general force field: A force field for drug-like molecules compatible with the CHARMM all-atom additive biological force fields. J Comput Chem 31:671-690.

18. Wu EL, Cheng X, Jo S, Rui H, Song KC, Dávila-Contreras EM, Qi Y, Lee J, Monje-Galvan V, Venable RM, Klauda JB, Im W (2014) CHARMM-GUI Membrane Builder toward realistic biological membrane simulations. J Comput Chem 35:1997-2004.
